# Computational modelling of dynamic recrystallisation of Ni-based superalloy during linear friction welding

**DOI:** 10.1007/s00170-021-08559-1

**Published:** 2022-01-12

**Authors:** Saviour I. Okeke, Noel M. Harrison, Mingming Tong

**Affiliations:** 1 Mechanical Engineering, School of Engineering, College of Science and Engineering, NUI Galway, Galway, Ireland; 2 I-Form, SFI Research Centre for Advanced Manufacturing, Dublin, Ireland; 3 Ryan Institute for Environmental, Marine and Energy Research, NUI Galway, Galway, Ireland

**Keywords:** Linear friction welding, Inconel 718, Dynamic recrystallisation, Gamma grain, Microstructural modelling, Multiphysics modelling

## Abstract

Linear friction welding (LFW) is an advanced joining technology used for manufacturing and repairing complex assemblies like blade integrated disks (blisks) of aeroengines. This paper presents an integrated multiphysics computational modelling for predicting the thermomechanical-microstructural processes of IN718 alloy (at the component-scale) during LFW. Johnson–Mehl–Avrami-Kolmogorov (JMAK) model was implemented for predicting the dynamic recrystallisation of *γ* grain, which was coupled with thermomechanical modelling of the LFW process. The computational modelling results of this paper agree well with experimental results from the literature in terms of *γ* grain size and weld temperature. Twenty different LFW process parameter configurations were systematically analysed in the computations by using the integrated model. It was found that friction pressure was the most influential process parameter, which significantly affected the dynamic recrystallisation of *γ* grains and weld temperature during LFW. The integrated multiphysics computational modelling was employed to find the appropriate process window of IN718 LFW.

## Introduction

Linear friction welding (LFW) is an advanced energy-efficient solid-state joining technology, which has important application in the manufacture of critical engineering components such as in the aerospace industry. LFW can produce high-quality welds by rapidly oscillating one workpiece relative to another while applying large compressive pressure. During LFW, friction heat is generated between the oscillating and stationary workpieces, leading to material softening and bonding of workpieces under sustained pressure. Unlike conventional fusion welding, the contacting surfaces of workpieces are welded together without remelting of the surfaces during LFW. LFW can join similar and dissimilar materials and a wide range of materials have been successfully joined, such as titanium alloys, aluminium alloys, and steels, with the foremost industrial application in aeroengine alloys of blade integrated disks (blisks) [1–8]. Despite the increasing application of LFW, the complex interaction between heat transfer, deformation of weld and material microstructural evolution during LFW is not well understood.

Inconel 718 (IN718) is one of the Ni-based superalloys, which has excellent high-temperature strength, strong oxidation resistance, and high corrosion resistance. It has been widely applied in modern aero-engines, steam turbine power plants, nuclear power systems, and marine and oil applications [8–11]. In precipitation strengthened IN718, the primary (*γ*) phase and secondary (*γ'*, *γ"* and *δ*) have a direct influence on mechanical properties of the alloy. *γ"* (Ni_3_Nb) and *γ'* (Ni_3_Al) are the primary and secondary strengthening precipitates, respectively. The *δ* phase has the same composition as the *γ"* phase, precipitates at the grain boundaries, and can prevent grain boundary migration [12–14]. These primary and secondary phases of IN718 undergo significant microstructural change during thermomechanical processing, due to elevated temperature and significant material deformation, which can significantly affect the mechanical properties of manufactured components.

Several studies have reported the influence of thermomechanical processes on the microstructural properties and resultant mechanical behaviour of IN718 during LFW [11, 15–18]. For instance, it was found that the level of friction heat generated at the friction interface of the weld could directly determine the local hardness profile (as well as the *γ* grain size and *γ'* volume fraction) of an IN718 LFW weld joint [11, 18]. Markov et al. used the Maxwell visco-elastic model for predicting the thermomechanical processes of steel, which is not popularly used for computational modelling of IN718 LFW like the strain-compensated Arrhenius equation in Qin et al. [19, 20]. The dynamic recrystallisation of *γ* grains during LFW might refine material microstructure and increase the hardness and tensile strength of weld [20–23]. In some studies, the hardness profile at the friction interface was attributed to a combination of variation in *γ* grain size, *γ'* volume fraction, size and distribution, *γ*–*γ'* misfit, and work hardening due to residual plastic work [23–26]. Mary and Jahazi noted that dynamic recrystallisation and dynamic recovery of *γ* grains occur simultaneously with the loss of the *δ* phase in IN718 weld joint during LFW [16]. They reported that *γ* grains within $\pm$ 1 mm distance from the friction interface were three times smaller than those of the parent material (16 μm) because DRX occurred during LFW [16]. In another study, Mary and Jahazi observed that beyond 3-mm distance from the friction interface, the *γ* grain size remains constant and equal to the *γ* grain size of the base metal (non-welded) material and the temperature is below the *δ* phase equilibrium solvus temperature (~ 1283 K) [17]. Similarly, Chamanfar et al. observed fine *γ* grain size of 7.5 μm within 0.9 mm of the friction interface of linear friction welded Waspaloy, whereas the parent material grain size was 15.1 μm [25]. Similar grain refinement at the friction interface zone was reported for inertia friction welding of RR®1000 Ni-based superalloy [27, 28]. Li et al. [29] found that the presence of *γ'* phase can hinder DRX; hence, the occurrence of DRX is usually observed in the region where intergranular and intragranular *γ'* are dissolved. Overall, the recrystallised *γ* grain size has been consistently recognised as an important factor in refining material microstructure of the weld and directly determining the hardness and tensile strength of weld [30–32]. In order to optimise the design of LFW process parameters, the influence of thermomechanical processes on the microstructural evolution of IN718 needs to be systematically and quantitatively analysed in relation to the DRX of *γ* grain during LFW process.

LFW process optimisation can be achieved by experimentally varying three important welding parameters of such as friction pressure, oscillating frequency, and oscillating amplitude. Multiple researchers have reported the influence of different LFW parameter configurations on such as weld temperature, heating rate, and resultant microstructure and mechanical properties of IN718 weld [20, 33–35]. Ma et al. [18] found that DRX and dynamic recovery (DRV) could be enhanced by increasing the friction pressure and oscillating amplitude of LFW. Similar results of enhanced DRX were reported in other research studies [36–38]. Geng et al. noted that the high plastic flow stresses of IN718 at high temperatures were related to the undeformed morphology of numerous refined grains in the thermomechanically affected zone (TMAZ) of IN718 weld [11]. Chamanfar et al. [8], Geng et al. [11] and Masoumi et al. [36] have shown in different studies that grain size is dependent on temperature and strain rate, which are determined by friction pressure, oscillating frequency, and oscillating amplitude [8, 11, 36]. However, temperature and strain rate, as well as their influence on grain size, are difficult to measure during LFW using experimental methods because LFW is a very dynamic process, which involves very rapid relative motion of workpieces under high pressure [8, 11, 36]. Several experimental studies on LFW used indirect measurement techniques (e.g. infrared thermal imaging, thermocouple) to estimate weld temperature, strain rate, plastic strain, and stress distribution because direct measurement of LFW process is relatively difficult [8, 36]. Xie et al. [39] used a deformation-driven metallurgy method to sinter an aluminium maxtrix composite reinforced by graphene nanoplatelets, during which the friction heat and plastic deformation caused significant DRX of aluminium grains.

Computational modelling methods can effectively predict thermomechanical processes as well as material microstructural evolution during materials processing. Such methods have been widely researched and published particularly for the thermomechanical processes of IN718 LFW [20, 33, 35, 40–45]. However, there has been very little work published in relation to the computational modelling for the material microstructural evolution during IN718 LFW. Computational modelling studies about DRX processes have been published for steel, aluminium alloys, and titanium alloys during hot forging or hot isothermal compression testing processes [30, 46–52]. While these studies have used renowned models such as the Johnson–Mehl–Avrami–Kolmogorov (JMAK) model and the cellular automaton model, they have not been implemented for modelling of DRX of *γ* grains during LFW of IN718 (or any nickel-based superalloy) [30, 46, 51, 53, 54]. The authors previously developed an integrated multiphysics computational model for predicting the microstructural evolution of *δ* phase during LFW of IN718 at the component scale [34]. By using a similar concept of multiphysics computational modelling, a new integrated computational modelling was developed for predicting the DRX of *γ* grains during LFW of IN718. In Sect. 2 of this paper, the thermomechanical model for the material response, the microstructural model for the DRX of *γ* grains, and their coupling for LFW modelling of IN718 are presented.

## Modelling method

The integrated multiphysics computational modelling was developed by sequentially coupling a thermomechanical submodel for the LFW process to a microstructural submodel for DRX of *γ* grains in IN718 alloy. The overall integrated model was implemented in two-dimensional (2D) computational modelling for IN718 LFW by using finite element software package ABAQUS in conjunction with a custom written user-hardening subroutine (VUHARD) [55].

### Thermomechanical model

#### Set-up of thermomechanical model

**Fig. 1:**
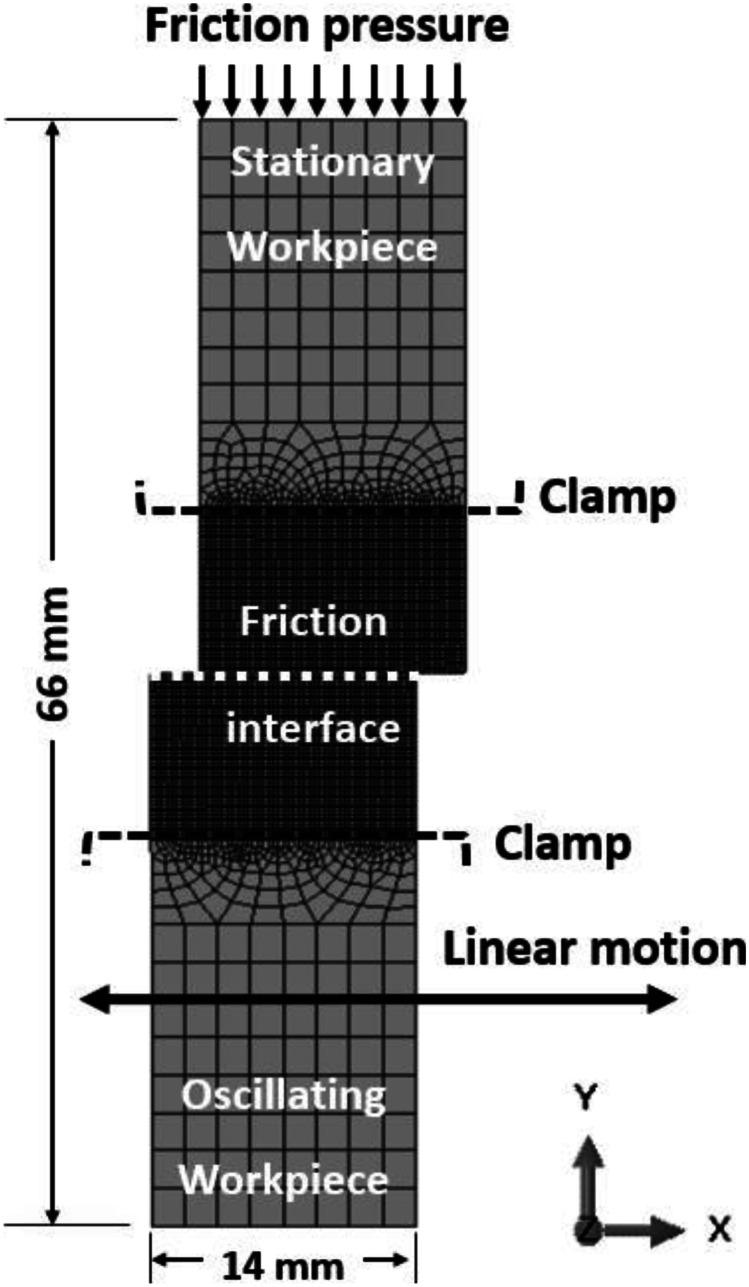
Setup of the 2D simulation domain as well as computational mesh of the friction-paired deformable workpieces

The thermomechanical model for LFW of IN718 was implemented by using dynamic temperature-displacement analysis in the Abaqus/Explicit solver, which is suitable for resolving contact problems as well as overcoming excessive element distortion by dynamic remeshing [55]. Similar thermomechanical modelling was previously presented by the authors, in which comprehensive presentations of the thermal and mechanical sub-models of IN718 during LFW can be found in [33, 34]. The thermal sub-model and mechanical sub-model are fully coupled. The LFW process lasts for 5.0 s of welding time. The geometry and mesh of the simulation domain are schematically shown in Fig. 1, which consist of a top (oscillating) workpiece and a bottom (stationary) workpiece, contacting each other at the friction interface as a deformable friction pair. The workpieces were discretised using the deformable plane strain formulation with elements defined in the *X*–*Y* plane and restricting deformation only to the defined plane. Both workpieces have the same dimension of 33 mm by 14 mm, and the computational domain of each workpiece comprises two structured mesh zones and one unstructured mesh zone. The workpiece dimensions were taken from Geng et al. and Qin et al. and their experimental data (corresponding to LFW setup J13 in this paper) was used by the authors for model verification [11, 20].

In this study, element types of CPE4RT and CPE3T (4-node and 3-node thermally coupled, displacement and temperature, reduced integration, hourglass control) were specified on the 2D model. A fine mesh of element size ~ 0.3 mm was used within 10-mm distance from the friction interface, in the region where high temperature and high plastic deformation happens. Coarse mesh of size ~ 1.8 mm was used in the region of the workpieces beyond 10 mm of the friction interface, where relatively low temperature and low plastic deformation happens. A mesh convergence study for the thermomechanical model has been presented previously by the authors in [33, 34]. There are 5978 elements and 6170 nodes for the entire simulation domain at the start of computation. To control excessive distortion of computational mesh domain during material softening and extrusion of LFW process, the arbitrary Lagrangian–Eulerian (ALE) adaptive dynamic remeshing was implemented for automatic solution mapping in the Abaqus/Explicit solver [55]. The semi-automatic mass scaling criterion was specified using a constant value of 800 at the beginning of each dynamic temperature-displacement explicit analysis step [55, 56]. This semi-automatic mass scaling criterion is commonly used because it limits the kinematic energy to less than 5% of the internal energy as well as reduces computational cost [6, 40, 41].

#### Thermal and mechanical behaviour

The thermomechanical model of this study mathematically formulates the thermal and mechanical responses of IN718 material. The heat diffusion equation is expressed as [20, 42, 57]:

1 $$\rho C_p\frac{\partial T}{\partial t}=\nabla\cdot\lambda\nabla T+{\eta{\overline\sigma}\;\dot{\overline\varepsilon}}^{pl}$$

where $\rho$ is material density in kgm^−3^, ${C}_{p}$ is specific heat capacity in Jkg^−1^ K^−1^, $\lambda$ is temperature-dependant thermal conductivity in Wm^−1^ K^−1^, $T$ is temperature in Kelvin, and $t$ is time in seconds. $\eta$, $\tilde{\sigma }$, and ${\dot{\overline{\varepsilon }}}^{pl}$ are efficiency of converting mechanical energy to heat energy, effective stress in MPa, and plastic strain rate in s^−1^, respectively. Mechanical energy efficiency $\eta$ is specified as 0.9 and the inelastic heat fraction is set to 0.9. During LFW, interfacial frictional heat is conducted through the contacting surfaces, while convective and radiative heat losses (to the ambient environment) occur simultaneously. The thermal boundary condition is described as:

2 $$\lambda \frac{\partial T}{\partial n}=\eta {\tau }_{fr}{\nu }_{s}- {h}_{k}\left({T}_{w}- {T}_{c}\right)- \theta \phi \left({T}_{w}^{4}-{T}_{c}^{4}\right)$$

where ${\tau }_{fr}$, ${\nu }_{s}$, ${h}_{k}$, ${T}_{w}$, and ${T}_{c}$ are friction stress in MPa, slip velocity mm·s^−1^, convective heat transfer coefficient in Wm^−2^ K^−1^, wall (boundary) temperature in Kelvin, and ambient temperature in Kelvin, respectively. $\theta$, $\phi$, and $n$ are Stefan–Boltzmann constant in Wm^−2^ K^−4^, emissivity and workpiece surface area in m^2^. Heat transfer coefficient was specified as a fixed value of 100 Wm^−2^ K^−1^ [20, 35, 42]. The thermal properties and boundary conditions presented in this paper are the same as those presented by the authors in a past paper [34].

Mechanical behaviour of the material during LFW is governed by the equilibrium equation expressed as [20, 35]:

3 $$\rho \frac{{\partial }^{2}u}{\partial {t}^{2}}= \nabla \left(\sigma \right)+ \rho F$$

where $u$ is material displacement vector, $\sigma$ is stress tensor, $F$ is body force vector per unit mass, and $t$ is time. The classic von Mises generalised model of the rate-dependent material is:

4 $$f\left(\sigma ,\varphi \right)={\sigma }_{eq}-{\sigma }_{s}\left({\overline{\varepsilon }}^{pl}, {\dot{\overline{\varepsilon }}}^{pl}, T\right)=0$$

where ${\sigma }_{eq}$ and ${\sigma }_{s}$ are effective stress and material yield stress, respectively. The mechanical boundary condition is pressure uniformly applied at the top surface of the stationary (i.e. top) workpiece. This surface has constrained *x*-axis displacement and unconstrained *y*-axis displacement to permit axial shortening (see Fig. 1). The oscillating (i.e. bottom) workpiece has *x*-axis sinusoidal displacement, while displacement is constrained in the *y*-axis. The *x*-axis sinusoidal displacement of the oscillating workpiece is controlled by:

5 $$x={A}_{0}\;\mathrm{sin}\;2\pi {f}_{0}t$$

where ${A}_{0}$ is the amplitude of oscillation (mm), ${f}_{0}$ is the frequency of oscillation (Hz) and *t* is the instantaneous weld time, from 0.0 s to 5.0 s.

#### Constitutive material model and friction law

The elastic response of IN718 is assumed to be governed by Hooke’s Law. The constitutive material model employed in this study is the strain-compensated Arrhenius model expressed as [20, 35, 41]:

6 $$\begin{aligned}{\sigma }_{y}=\frac{1}{\alpha (\varepsilon )}\mathrm{ln}\left\{{\left[\frac{\dot{\varepsilon }}{A(\varepsilon )}\mathrm{exp}\left(\frac{Q(\varepsilon )}{RT}\right)\right]}^{\frac{1}{n(\varepsilon )}} \right. \\ + \left.{ \left[{ \left[\frac{\dot{\varepsilon }}{A(\varepsilon )}\mathrm{exp}\left(\frac{Q(\varepsilon )}{RT}\right)\right]}^{\frac{2}{n(\varepsilon )}}+1 \right]}^\frac{1}{2}\right\}\end{aligned}$$

where ${\sigma }_{y}$ is yield stress, $\dot{\varepsilon }$ is strain rate, $T$ is absolute temperature, $R$ is universal gas constant, $Q(\varepsilon )$ is the deformation activation energy, and $\alpha (\varepsilon )$, $n(\varepsilon )$, and $A(\varepsilon )$ are material constants. They are respectively expressed as polynomial functions of deformation strain as:

7 $$\begin{aligned}&\alpha\left(\varepsilon\right)=B_0+B_1\varepsilon+B_2\varepsilon^2+B_3\varepsilon^3+B_4\varepsilon^4+B_5\varepsilon^5\\&n\left(\varepsilon\right)=C_0+C_1\varepsilon+C_2\varepsilon^2+C_3\varepsilon^3+C_4\varepsilon^4+C_5\varepsilon^5\\&Q\left(\varepsilon\right)=D_0+D_1\varepsilon+D_2\varepsilon^2+D_3\varepsilon^3+D_4\varepsilon^4+D_5\varepsilon^5\\&ln\;A\left(\varepsilon\right)=F_0+F_1\varepsilon+F_2\varepsilon^2+F_3\varepsilon^3+F_4\varepsilon^4+F_5\varepsilon^5\end{aligned}$$

The coefficients of polynomial functions for the alloy material can be found in the research [40].

In this study, friction behaviour is represented by a plastically deformable friction pair implemented by using the ‘surface-to-surface explicit’ friction contact behaviour. The magnitude of contact pressure was computed in the thermomechanical modelling during LFW. Penalty tangential workpiece interaction was specified for the transmission of shear stresses across the contacting surfaces [7, 34]. The friction coefficient depends on sliding velocity, friction interface temperature, and contact pressure. A modified Coulomb’s friction law has been previously employed for the target alloy IN718 given as [41]:

8 $$\mu =a{{p}_{f}}^{b} {T}^{c}\;\mathrm{ exp}(d{v}_{s})$$

where ${p}_{f}$ is contact friction pressure, ${v}_{s}$ is sliding velocity, and $T$ is interface temperature between the contacting friction surfaces. The constants $a, b, c,\mathrm{ and }d$ are specified as 0.12, $-$ 0.233, 0.471, and $-$ 0.739, respectively [41]. Maximum frictional stress ${\tau }_{fr}$ is limited by the strain rate and temperature-dependent yield stress ${\sigma }_{y}$ of IN718 material expressed as [20, 41]:

9 $${\tau }_{fr}=\mathrm{min}\left(\mu {p}_{f}, \frac{{\sigma }_{y}}{\sqrt{3}}\right)$$

### Microstructural model for DRX of γ grain during LFW

LFW typically involves a very high heating rate and high weld temperature within a relatively short time, which may significantly affect the size of primary *γ* grains due to dynamic recrystallisation [17]. In this study, the DRX of *γ* grains during LFW was formulated by using the empirical JMAK model [30, 46, 51]. The volume fraction of recrystallised *γ* grains ${X}_{DRX}$ is given as [30]:

10 $$\begin{aligned}{X}_{DRX}=&\;1-\mathrm{exp}\\&\left[-\mathrm{ln}\;2{\left(\frac{\varepsilon -{\varepsilon }_{c} }{{\varepsilon }_{0.5}- {\varepsilon }_{c}}\right)}^{{n}_{d}}\right]\quad ; \quad\left(\varepsilon \ge {\varepsilon }_{c}\right)\end{aligned}$$

where $\varepsilon$ is current plastic strain, ${\varepsilon }_{c}$ is critical plastic strain for DRX initiation, ${\varepsilon }_{0.5}$ is strain for 50% volume fraction of DRX, and ${n}_{d}$ is the Avrami constant.

${\varepsilon }_{c}$ and ${\varepsilon }_{0.5}$ are expressed as [30]:

11 $${\varepsilon }_{c}={\varepsilon }_{c1}{Z}^{{n}_{c1}}$$

12 $${\varepsilon }_{0.5}={\varepsilon }_{b1}{Z}^{{n}_{b1}}$$

13 $$Z(\varepsilon )=\dot{\varepsilon }\mathrm{exp}\left[\frac{Q}{RT}\right]$$

where ${\varepsilon }_{c1}$, ${\varepsilon }_{b1}$, ${n}_{b1}$, ${n}_{c1}$ are material constants taken from [30]. Zener-Hollomon parameter $Z$ is expressed as the relation between weld temperature $T$ and plastic strain rate $\dot{\varepsilon }$ during LFW [46, 58]. Equation 10 follows a strain based DRX initiation criteria, such that DRX takes place when the current plastic strain reaches or exceeds the critical plastic strain. Geng et al. applied Eqs. 10–13 within the temperature range (1213–1453 K), which is higher than the *γ'* and *δ* equilibrium solvus temperatures of 1172 K and 1283 K [30, 34]. In this study, the critical temperature for onset of DRX is assumed to be 1213 K. The material constants in relation to DRX of *γ* grains of IN718 alloy were sourced from Geng et al. [11, 30].

${X}_{DRX}$ is predicted according to an incremental form of the JMAK model that is activated by plastic strain $\varepsilon$ expressed as [30, 46]:

14 $$\begin{aligned}{dX}_{DRX}=\;&\mathrm{exp}\left[-\mathrm{ln}\;2{\left(\frac{\varepsilon -{\varepsilon }_{c} }{{\varepsilon }_{0.5}- {\varepsilon }_{c}}\right)}^{{n}_{d}}\right]\\&\left[{n}_{d}\;\mathrm{ln}\;2{\left(\frac{\varepsilon -{\varepsilon }_{c} }{{\varepsilon }_{0.5}- {\varepsilon }_{c}}\right)}^{{n}_{d}-1}\right]\left(\frac{1 }{{\varepsilon }_{0.5}- {\varepsilon }_{c}}\right)d\varepsilon\end{aligned}$$

The volume fraction of recrystallised grains can be updated by using the equation [46, 58]:

15 $${X}_{{DRX}_{(t)}}= {X}_{{DRX}_{(t-1)}}+ {dX}_{{DRX}_{(t)}}$$

where ${X}_{{DRX}_{(t)}}$, ${X}_{{DRX}_{(t-1)}}$, and ${dX}_{{DRX}_{(t)}}$ are the current step volume fraction, previous step volume fraction, and increment in volume fraction of recrystallised grains, respectively. The size and dynamically recrystallised volume fraction of IN718 *γ* grains prior to DRX were set to be 20 μm and 0.01%, respectively [30]. The dynamically recrystallised grain size ${d}_{DRX}$ in μm can be computed using three different regimes of plastic strain rate based on Zener-Hollomon parameter $Z$ as follows [11]:

16 $${d}_{DRX}=-{g}_{l1}\mathrm{ln}\;Z+ {g}_{c1}\quad ; \quad\left(\dot{\varepsilon }<{0.1 s}^{-1}\right)$$

17 $${d}_{DRX}=-{g}_{l2}\mathrm{ln}\;Z+ {g}_{c2}\quad ;\quad\left({0.1 s}^{-1} <\dot{\varepsilon }<{10 s}^{-1}\right)$$

18 $${d}_{DRX}=-{g}_{l3}\mathrm{ln}\;Z+ {g}_{c3} \quad; \quad\left(\dot{\varepsilon }>{10 s}^{-1}\right)$$

where ${g}_{c1}$, ${g}_{c2}$, ${g}_{c3}$, ${g}_{l1}$, ${g}_{l2}$, and ${g}_{l3}$ are material constants, $\left(\dot{\varepsilon }<{0.1 s}^{-1}\right)$ for low strain rate regimes, $\left({0.1 s}^{-1} <\dot{\varepsilon }<{10 s}^{-1}\right)$ for medium strain rate regimes, and $\left(\dot{\varepsilon }>{10 s}^{-1}\right)$ for high strain rate regimes, and their values were taken from Geng et al. [11]. The average grain size ${D}_{ave}$ was calculated by using a weighted average of the recrystallised grain size ${d}_{DRX}$ and non-recrystallised grain size ${D}_{{ave}_{(t-1)}}$ as [46]:

19 $${D}_{{ave}_{(t)}}={d}_{DRX} \left({X}_{DRX}\right)+{D}_{{ave}_{(t-1)}} \left(1- {X}_{DRX}\right)$$

where $(t)$ and $(t-1)$ are the current and previous computational time steps.

### Model integration

**Fig. 2:**
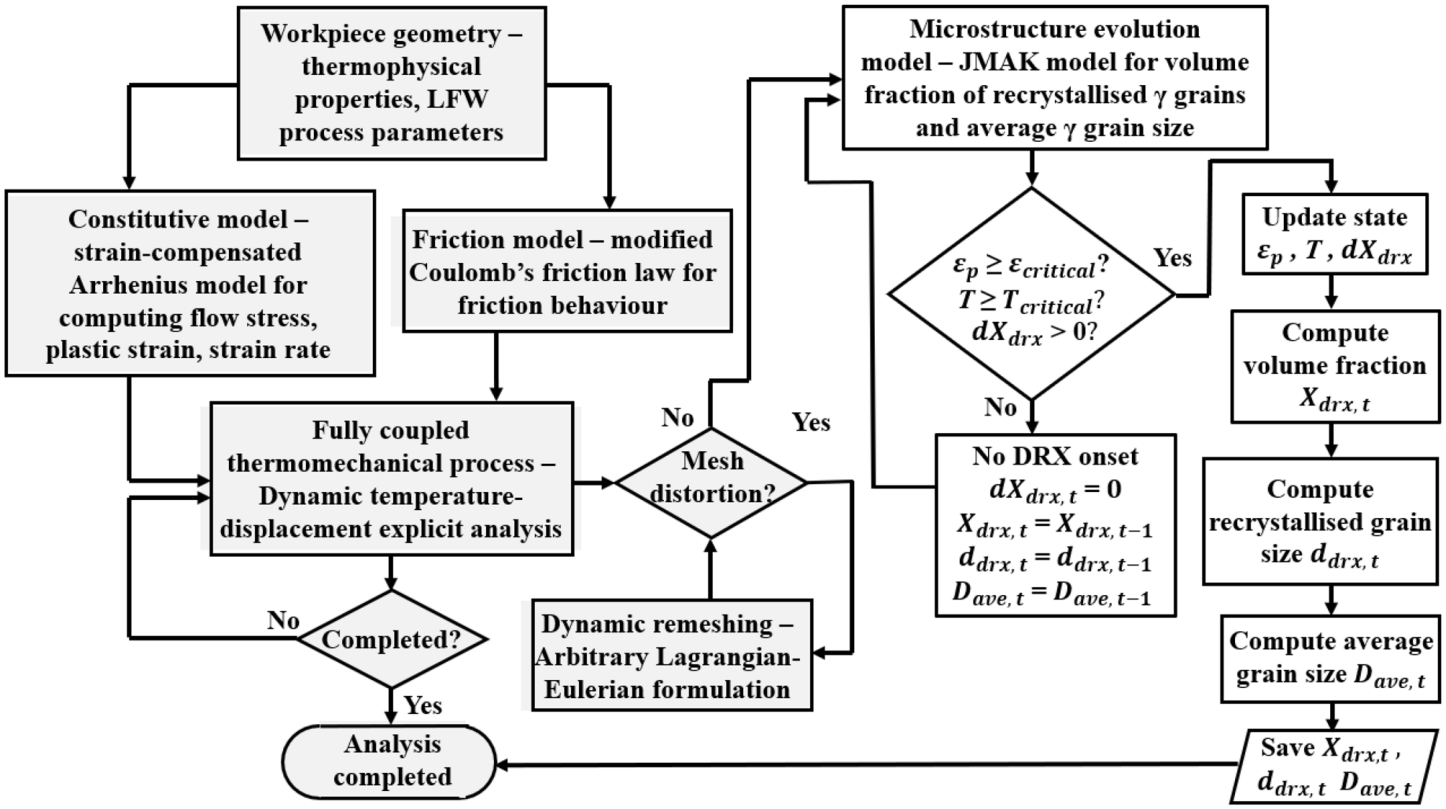
Flowchart of the integrated multiphysics computational modelling for LFW. The grey-coloured shapes represent the thermomechanical submodel and the white-coloured shapes represent the microstructural submodel in the VUHARD subroutine

Figure 2 presents a flowchart showing how the integrated multiphysics computational modelling works as well as the sequential coupling between the thermomechanical submodel and the microstructural submodel. For each computational time step, the thermomechanical and microstructural submodels are solved simultaneously. State variables such as temperature, plastic strain, and strain rate are computed by Abaqus/Explicit solver during the explicit dynamic temperature-displacement coupled analysis of LFW process. The VUHARD subroutine captures the computational results of state variables for use in the microstructural model. Then, Eqs. 10–19 are solved solved by Abaqus/Explicit in conjunction with related Abaqus/Explicit VUHARD code. In this sequential coupling between the thermomechanical sub-model and the microstructural sub-model, it is assumed that the DRX process of *γ* grains does not influence the thermomechanical responses of IN718 during LFW.

### Process parameters of LFW and material properties

In total, 20 different process parameter sets for LFW were defined (see Table 1) based on three key LFW process parameters, which are friction pressure ${p}_{f}$, oscillating amplitude ${A}_{0}$, and oscillating frequency ${f}_{0}$. The average rubbing velocity ${v}_{r}$ is determined from the oscillating amplitude ${A}_{0}$ and oscillating frequency ${f}_{0}$ using ${v}_{r}=4{A}_{0}{f}_{0}$ [1]. The 20 different LFW setups were used as the background of computational modelling for systematically predicting the influence of process parameters on weld temperature, *γ* grain size, and volume fraction of recrystallised *γ* grains during IN718 LFW.

**Table 1:** Process parameters applied in LFW computational modelling

Number of weld	Friction pressure *p*_*f*_ (MPa)	Oscillating frequency *f*_*0*_ (Hz)	Oscillating amplitude *A*_*0*_ (mm)	Average rubbing velocity *v*_*r*_ (mm/s)
J1	100	15	2.5	150
J2	100	20	2.5	200
J3	100	40	3.3	528
J4	200	20	3.3	264
J5	200	25	2.9	290
J6	200	40	2.5	400
J7	200	40	3.3	528
J8	300	15	2.5	150
J9	300	20	3.3	264
J10	300	25	2.9	290
J11	300	30	2.9	348
J12	400	15	2.5	150
J13	400	25	2.9	290
J14	400	20	3.3	264
J15	400	30	2.9	348
J16	500	20	3.3	264
J17	500	25	2.9	290
J18	500	30	2.5	300
J19	600	15	2.5	150
J20	600	20	2.5	200

The material properties of IN718 are assumed to be isotropic. The IN718 thermophysical properties used in the model were taken from [20]. Other material properties and LFW input process parameters can be found in Table 2. The chemical composition of IN718 is Ni-0.5Al-19.0Cr-18.5Fe-3.0Mo-5.1Nb-0.9Ti-0.04C in mass percentage [10].

**Table 2:** Other related process, material and modelling parameters used in the modelling [20]

*Thermomechanical input parameter*	*Value*
Room temperature (K)	298
Liquidus temperature of alloy (K)	1633
Thermal conductivity *λ* (W/m/K)	0.016* T* + 16.668
Specific heat capacity *c*_*p*_ (J/kg/K)	0.33* T* + 452.09
Expansion *α*_*w*_ (1/K)	4 $\times$ 10^−9^* T* + 10^−5^
Density *ρ* (kg/m^3^)	8420
Elastic modulus *E* (MPa)	221,000
Poisson’s ratio *ѵ*	0.3
Inelastic heat fraction	0.9
Heat partition coefficient	0.5
Friction energy change to heat	0.9
Mean friction coefficient	0.01─0.60
Shear stress limit (MPa)	60─100

The initial volume fraction of recrystallised *γ* grains before the start of LFW (which is also its lower limit) is set to 0.01% (for no recrystallised grains at all) and the upper limit of volume fraction of recrystallised *γ* grains is set to 99.99% (for fully recrystallised material). This will avoid numerical singularities, whilst having a very limited influence on the modelling results. Before the start of LFW, the size of non-recrystallised *γ* grains is assumed to be 20 μm and the size of recrystallised *γ* grain is assumed to be 3 μm.

## Results and discussion

### Temperature and plastic strain evolution

**Fig. 3:**
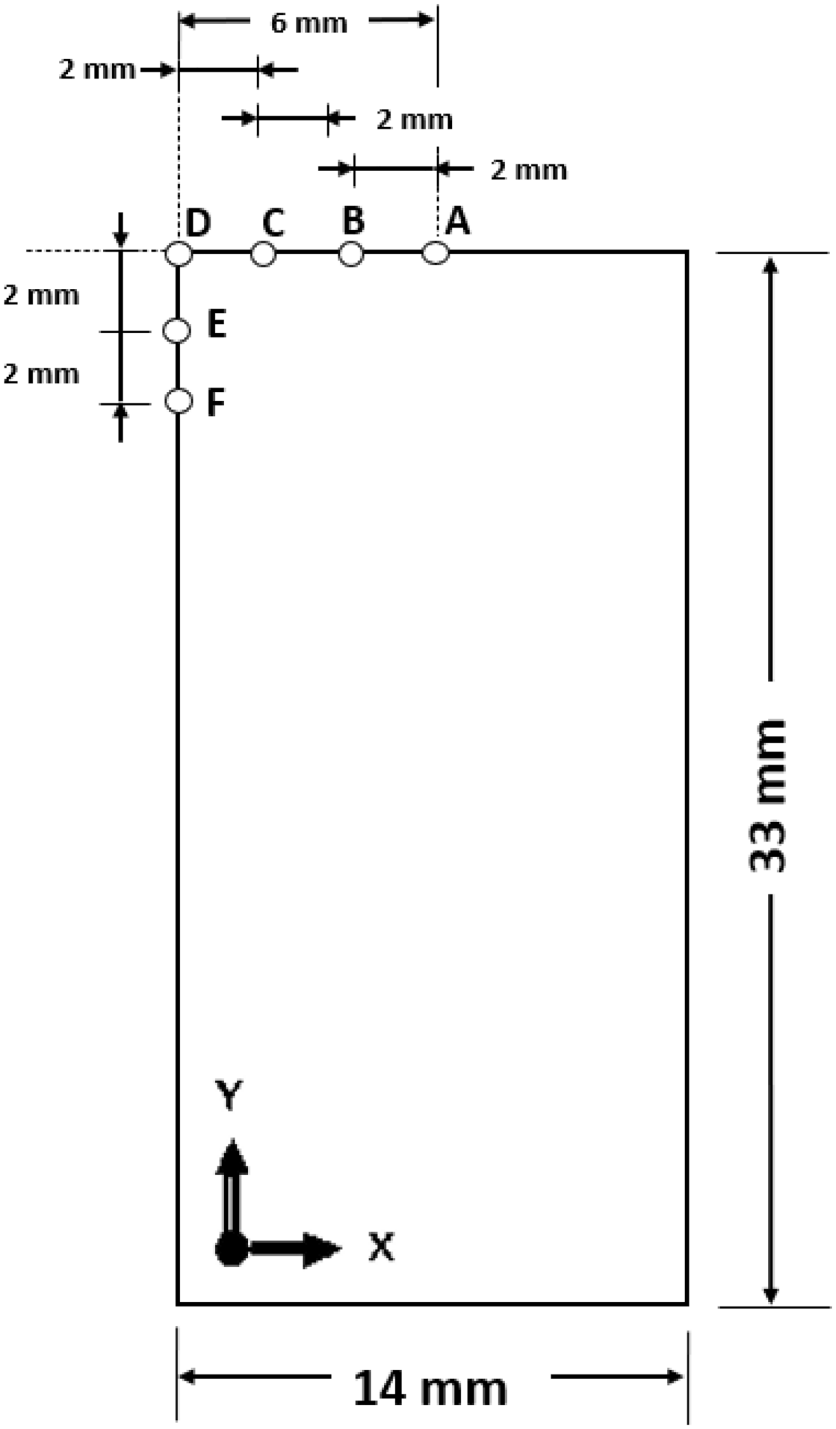
2D geometry of the bottom workpiece of LFW and the position of six sampling points (A, B, C, D, E, and F) identified on the friction interface and left side of the bottom workpiece.

Figure 3 shows six sampling points A, B, C, D, E, and F (not drawn to scale) on the friction interface and the left side of the bottom workpiece at the start of welding. The sampling points are 2 mm apart before welding starts and can get displaced further from one another during LFW because of the deformation of the weld. Line profiles of some modelling output parameters are presented for only the bottom workpiece considering that thermal histories and microstructural evolution are generally mirrored in both workpieces across the friction interface [11, 20, 35]. Thermomechanical model verification in relation to weld temperature, axial shortening, and flash shape has been previously presented by the authors in [33, 34]. Thus, this study focuses primarily on the analysis of the microstructural modelling results.

**Fig. 4:**
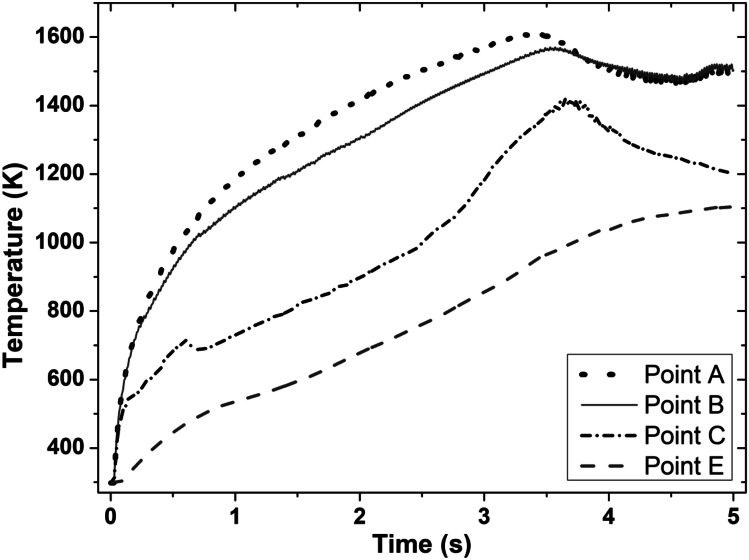
Temperature histories of sampling points A, B, C, and E on the bottom workpiece for LFW setup J13

**Fig. 5:**
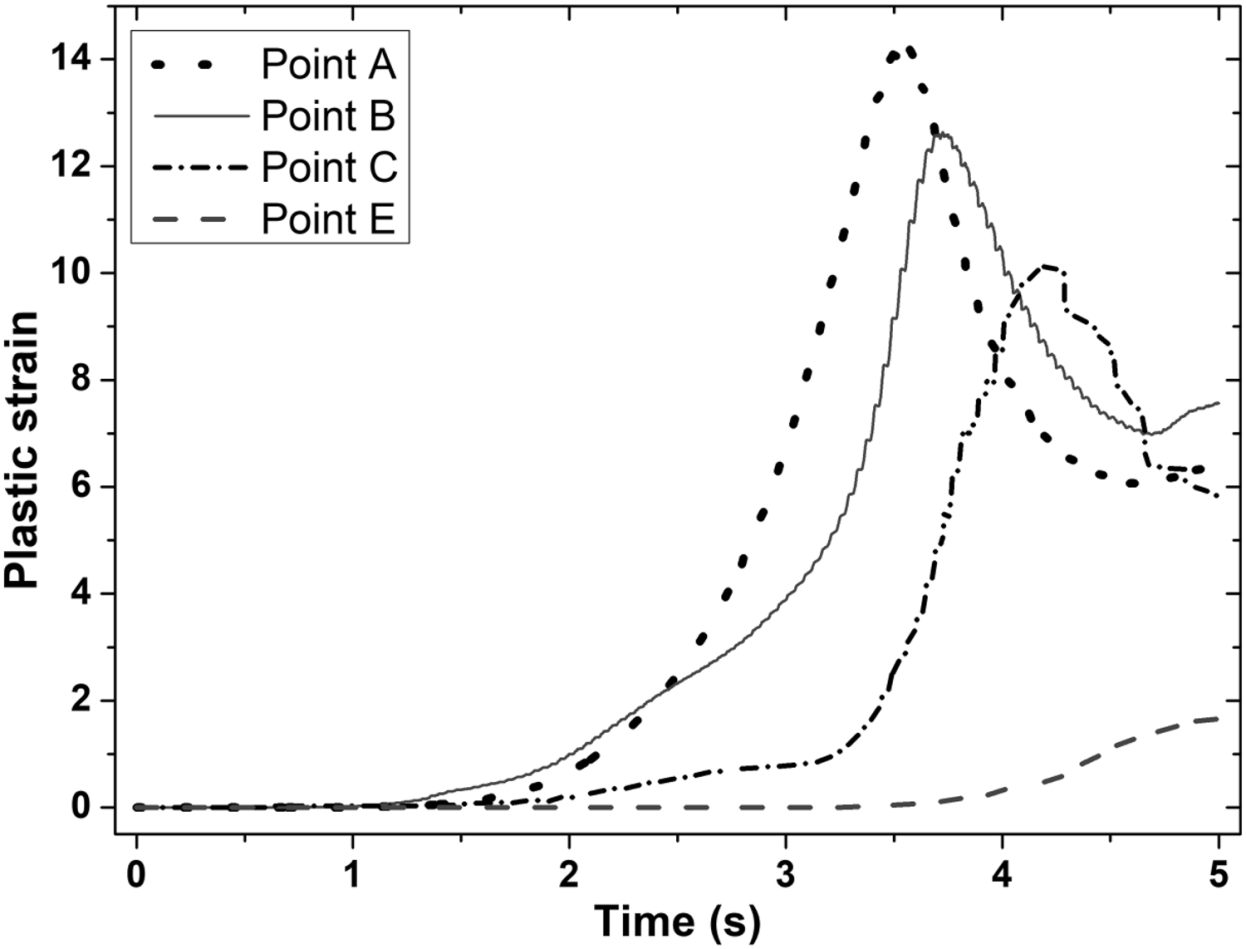
Evolution of plastic strain with time at sampling points A, B, C, and E on the bottom workpiece for LFW setup J13

Figures 4 and 5 show the computational modelling results of the evolution of temperature and plastic strain with time at sampling points A, B, C, and E (as shown in Fig. 3) for the LFW setup J13. LFW setup J13 was selected as the reference welding parameter set in this study to ensure consistent comparison with the experimental results of Geng et al. (based on the same workpiece dimension and weld configuration) and to achieve model verification [11]. Additionally, setups J1 to J20 were used to show other capabilities of the integrated computational model.

In Fig. 4, the maximum temperature at sampling points A, B, C, and E is 1610 K, 1570 K, 1420 K, and 1106 K, respectively. These maximum temperature levels are lower than the liquidus temperature of IN718 (~ 1633 K). At such elevated levels of temperature, the weld undergoes significant material softening, flash formation and extrusion at the friction interface of workpieces [8]. Sampling point A is shown to have the highest temperature; it is exactly at the mid-region of the friction interface. Sampling points B, C, and E show lower levels of temperature compared to sampling point A and these three points are further away from the mid-region of the friction interface. The maximum temperature was reached at 3.55 s, 3.53 s, and 3.65 s of welding for sampling points A, B, and C, respectively, while the maximum temperature was reached at 5.00 s of welding for sampling point E. The evolution of temperature with time at different stages of the LFW process was well explained in the previous work of the authors [33, 34]. Although temperature levels vary at different positions on the workpiece, it is the highest at the centre of the friction interface and becomes increasingly lower away from the centre [20, 33].

In Fig. 5, the maximum plastic strain at sampling points A, B, C, and E is 14.29, 12.64, 10.14, and 1.66, respectively, which was reached at welding time 3.53 s, 3.73 s, 4.21 s, and 5.00 s, respectively. These maximum plastic strains were reached in the equilibrium and extrusion stages of LFW, as explained in the author’s previous work [33]. However, only sampling points A, B, and C are displaced considerably. Similar to the varying levels of temperature, the plastic strain level varies at different positions on the bottom workpiece. It reached a maximum level at the centre of the friction interface and becomes increasingly lower away from the centre. Other researchers found similar variations in the plastic strain at different regions of the workpieces during LFW [20, 35, 42]. The temperature histories and plastic strain evolution (see Figs. 4 and 5), as well as strain rate, are important thermomechanical modelling results, which are used as inputs of microstructural modelling of DRX process of *γ* grains during LFW.

### Microstructural model verification and evolution of γ grains

**Fig. 6:**
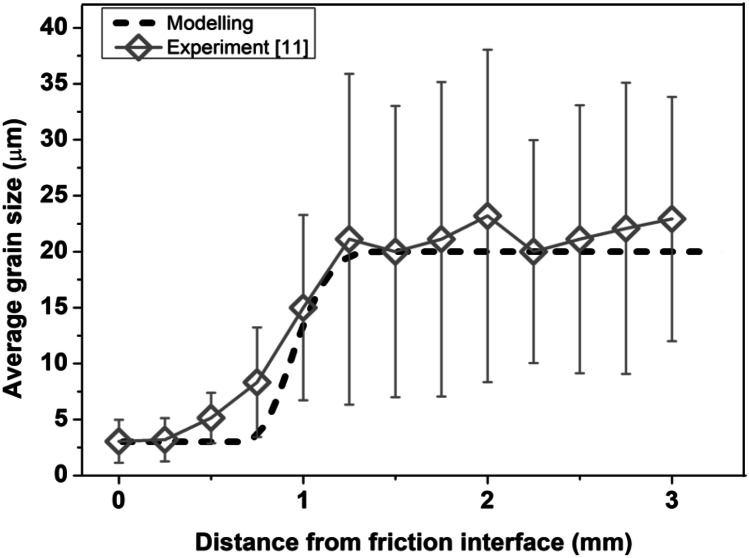
Comparison of the experimentally measured result of average *γ* grain size (experiment) of Geng et al. [11] with computational modelling results of average *γ* grain size (modelling) in this study for path L─M of LFW setup J13 at 5.0 s of welding

In order to verify the integrated model in terms of modelling the DRX of *γ* grains during LFW of IN718, the computational modelling results of this paper are compared with related experimental results of Geng et al. as shown in Fig. 6 [11]. Specifically, the LFW of IN718 was completed for 5 s in the modelling of the authors as well as in the experimental research of Geng et al. according to the setup J13 [11]. The average size of *γ* grain is characterised along a sampling path L─M of the weld. It can be seen in Fig. 6 that the modelling result of average *γ* grain size agrees with related experimental results, especially beyond 1-mm distance from the friction interface. This proves that the integrated computational model reasonably predicted the evolution of *γ* grain size due to DRX during LFW of IN718. Besides verifying the model using LFW setup J13, other capabilities of the integrated computational model are shown using LFW setups J1 to J20 in subsequent sections of this paper.

**Fig. 7:**
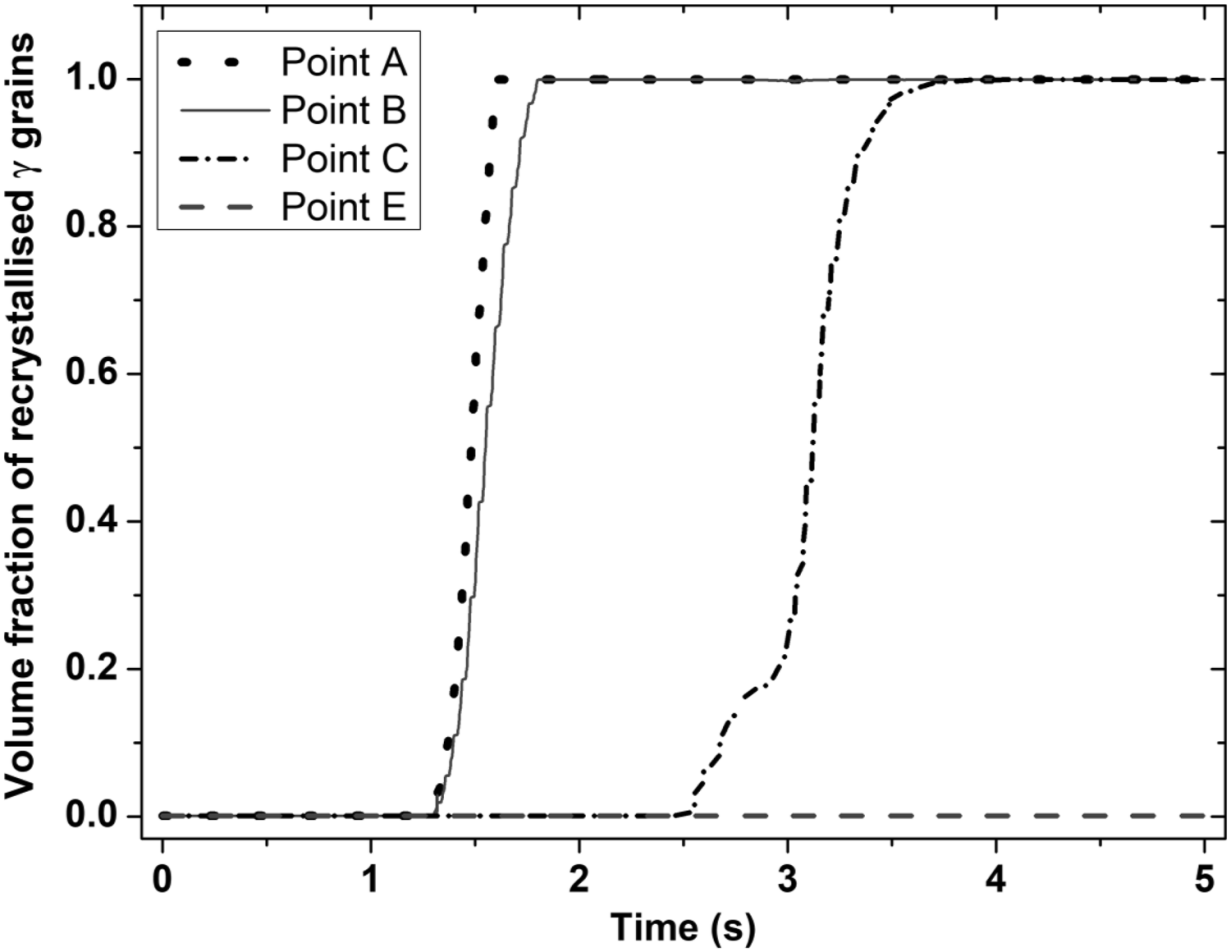
Temporal evolution of volume fraction of recrystallised *γ* grains at sampling points A, B, C, and E of the bottom workpiece for LFW setup J13

**Fig. 8:**
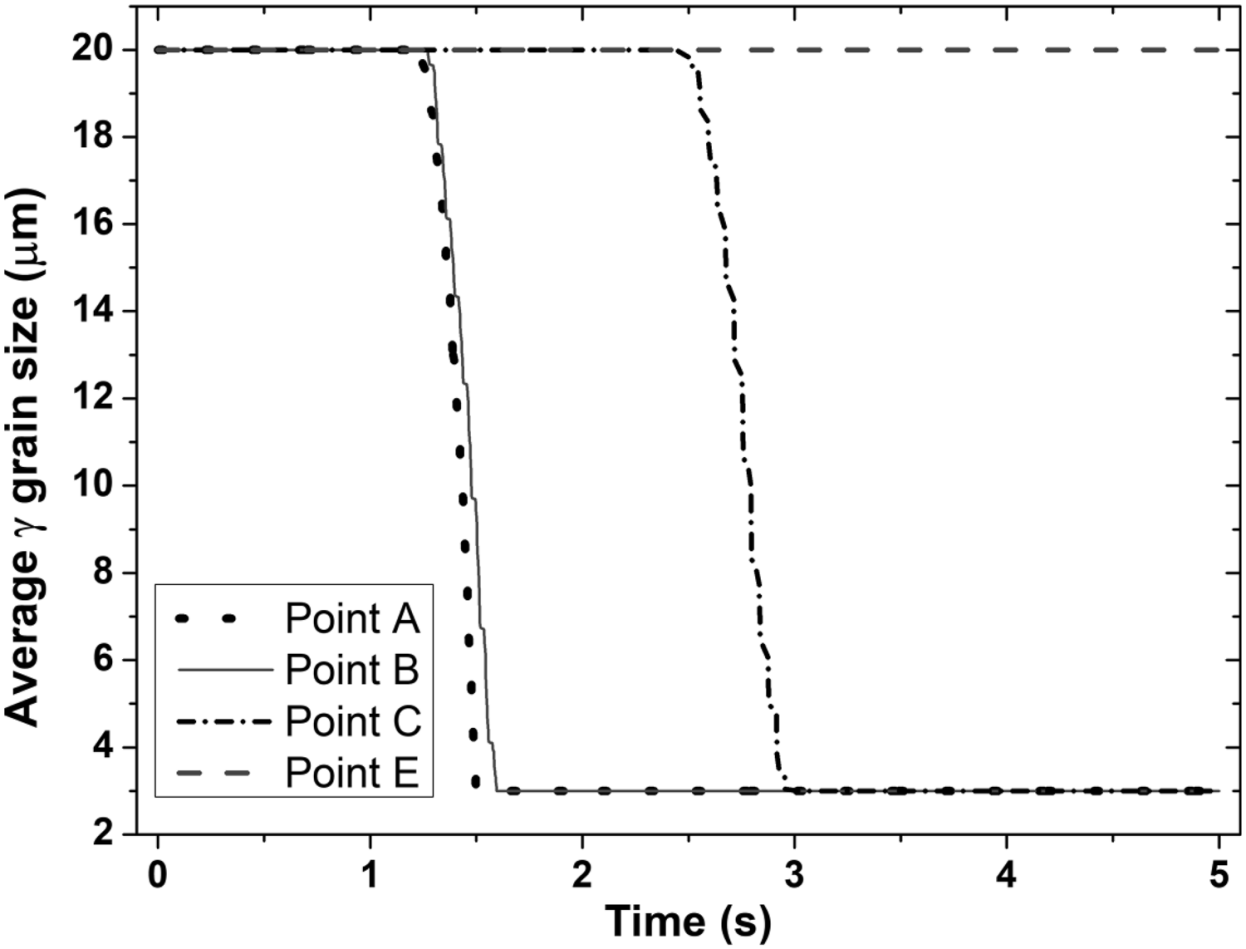
Temporal evolution of average *γ* grain size at sampling points A, B, C, and E of the bottom workpiece for LFW setup J13

Figures 7 and 8 show the temporal evolution of volume fraction of dynamically recrystallised *γ* grains and the temporal evolution of average *γ* grain size at sampling points A, B, C, and E of the bottom workpiece for LFW setup J13. In Fig. 7, *γ* grains get fully recrystallised at welding time of 1.9 s, 2.7 s, and 3.4 s at sampling points A, B, and C, while the volume fraction of recrystallised *γ* grains evolves between 0.01 and 99.99%. Comparing Fig. 7 with Fig. 8, it can be seen that the increase in the volume fraction of recrystallised *γ* grains corresponds to a decrease in the average size of *γ* grains. It indicates that the DRX process refines *γ* grains during LFW of IN718 [30, 51, 59]. At sampling point E, the volume fraction of recrystallised *γ* grains as well as the average size of *γ* grains did not change during LFW because the computationally predicted maximum temperature of the material (1106 K) during LFW at sampling point E is below the critical temperature (1213 K) for the onset of DRX.

**Fig. 9:**
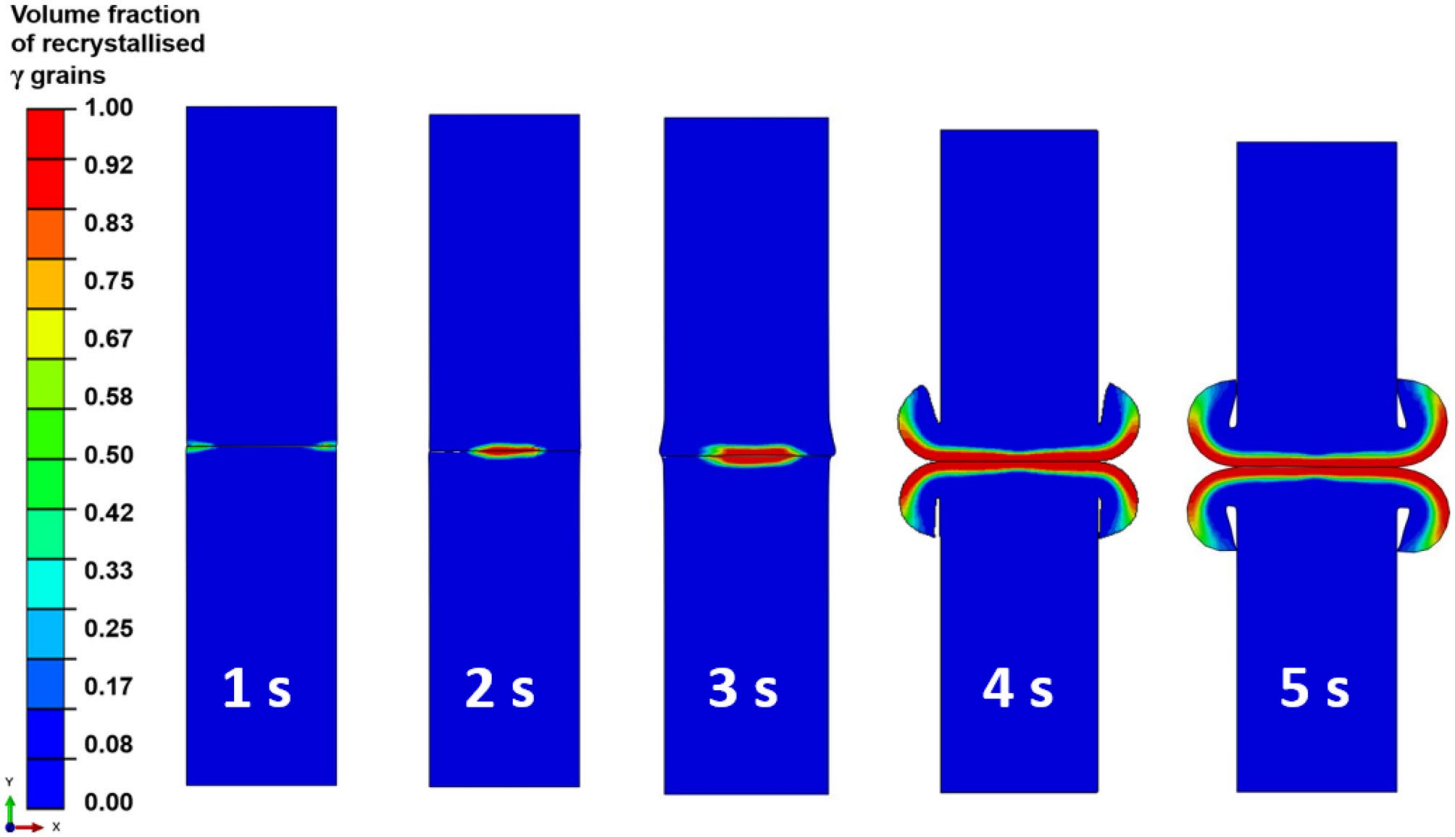
Temporal evolution of spatial distribution of volume fraction of recrystallised *γ* grains of the weld during LFW process for setup J13 where ${p}_{f}=400\mathrm{ MPa}, {f}_{0}=25\mathrm{ Hz},\mathrm{ and }{A}_{0}=2.9\mathrm{ mm}$

Figure 9 shows the evolution of the spatial distribution of volume fraction of recrystallised *γ* grains during the LFW process, in the overall weld for setup J13. At 1.0 s of welding, the volume fraction has not changed in the weld because the maximum temperature is still below the critical temperature of 1213 K for the onset of DRX. At 2.0 s of welding, the maximum volume fraction has increased to 0.83, around the friction interface. Up to 3.0 s of welding time, the maximum volume fraction has further increased to 1.00 at the friction interface and all *γ* grains become recrystallised in the close vicinity of a very significantly deformed friction interface. The volume fraction is 1.00 up to 5.0 s of LFW. Overall, because high temperature and significant plastic deformation of material only happen in the close vicinity of the friction interface, full recrystallisation can only happen in a very narrow zone that is within 1.5 mm of each workpiece relative to the friction interface, which is approximately the red zone as shown in Fig. 9.

**Fig. 10:**
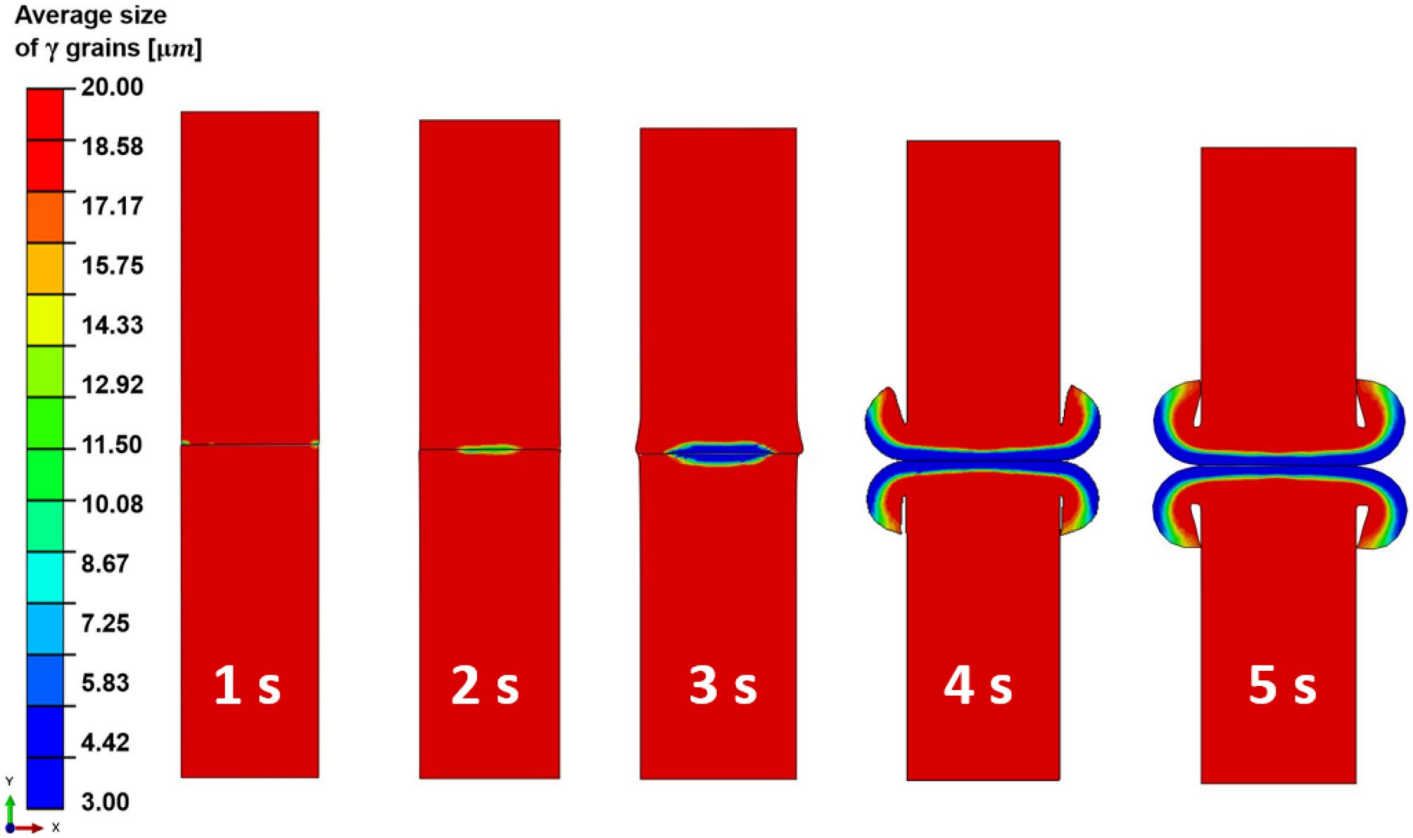
Temporal evolution of the spatial distribution of average *γ* grain size of the weld during LFW process for setup J13 where ${p}_{f}=400\mathrm{ MPa}, {f}_{0}=25\mathrm{ Hz},\mathrm{ and }{A}_{0}=2.9\mathrm{ mm}$

Figure 10 shows the temporal evolution of the spatial distribution of average *γ* grain size for setup J13. At 1.0 s of welding, the average grain size has not changed in the weld because the maximum temperature is below the critical temperature of 1213 K for the onset of DRX and DRX has not started yet. At 2.0 s of welding, the average *γ* grain size has decreased from 20.0 to 14.3 μm near the centre of the friction interface. It further decreases to 4.4 μm at the centre of friction interface at welding time of 3.0 s. At 4.0 s of welding, the average *γ* grain size is 3.0 μm at the friction interface, while *γ* grains are still relatively larger away from the friction interface. At 5.0 s of welding, the average *γ* grain size is 3.0 μm along the entire significantly deformed friction interface. However, such a fine grain region exists within 1.5 mm relative to the friction interface of each workpiece. Away from the friction interface, the average *γ* grain size continuously increases from 3.0 to 20.0 μm, as shown in Figs. 6 and 10.

By comparing the modelling results shown in Figs. 9 and 10, it can be seen that very significant dynamic recrystallisation very rapidly happened in the weld during LFW but only within a very limited volume of material that is within 1.5 mm relative to the friction interface. Within this region, *γ* grains become fully recrystallised since approximately 5.0 s of LFW. The fully recrystallised material has a relatively small average *γ* grain size.

### LFW process parameter optimisation

#### Relationship between average γ grain size, volume fraction of recrystallised γ grains, and temperature of weld

**Fig. 11:**
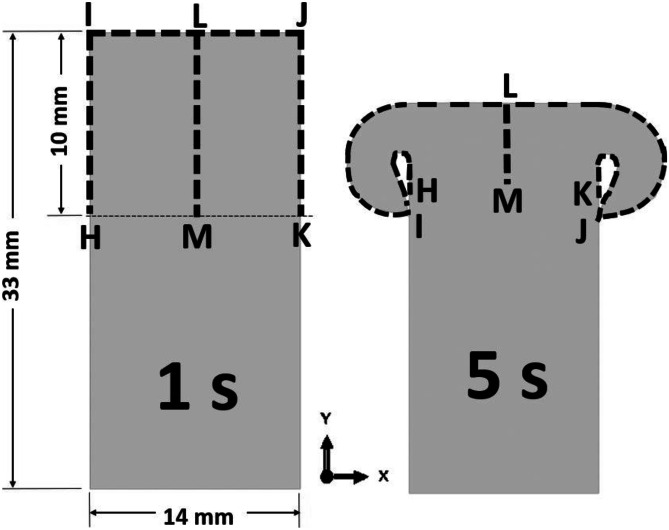
Data sampling paths L─M (10 mm long) and H─I─J─K (34 mm long) at the surface of the bottom weld based on LFW setup J13

The modelling results of such as volume fraction of recrystallised grains, average *γ* grain size, and weld temperature are analyzed along path L─M (10 mm long) and path H─I─J─K (34 mm long) as shown in Fig. 11, at the surface of the bottom workpiece based on LFW setup J13. There are 53 and 157 sampling points on paths L─M and H─I─J─K. Axial shortening and flash formation of weld can cause the length of path L─M to reduce considerably while the length of path H─I─J─K increases considerably during LFW process (see Fig. 11). The direction of path L─M in this paper is from L to M and that of path H─I─J─K is from H to I, I to J, and J to K. These paths are data sampling paths, which are employed in this paper only for data analysis purposes. They do not imply any partition of the workpiece.

**Fig. 12:**
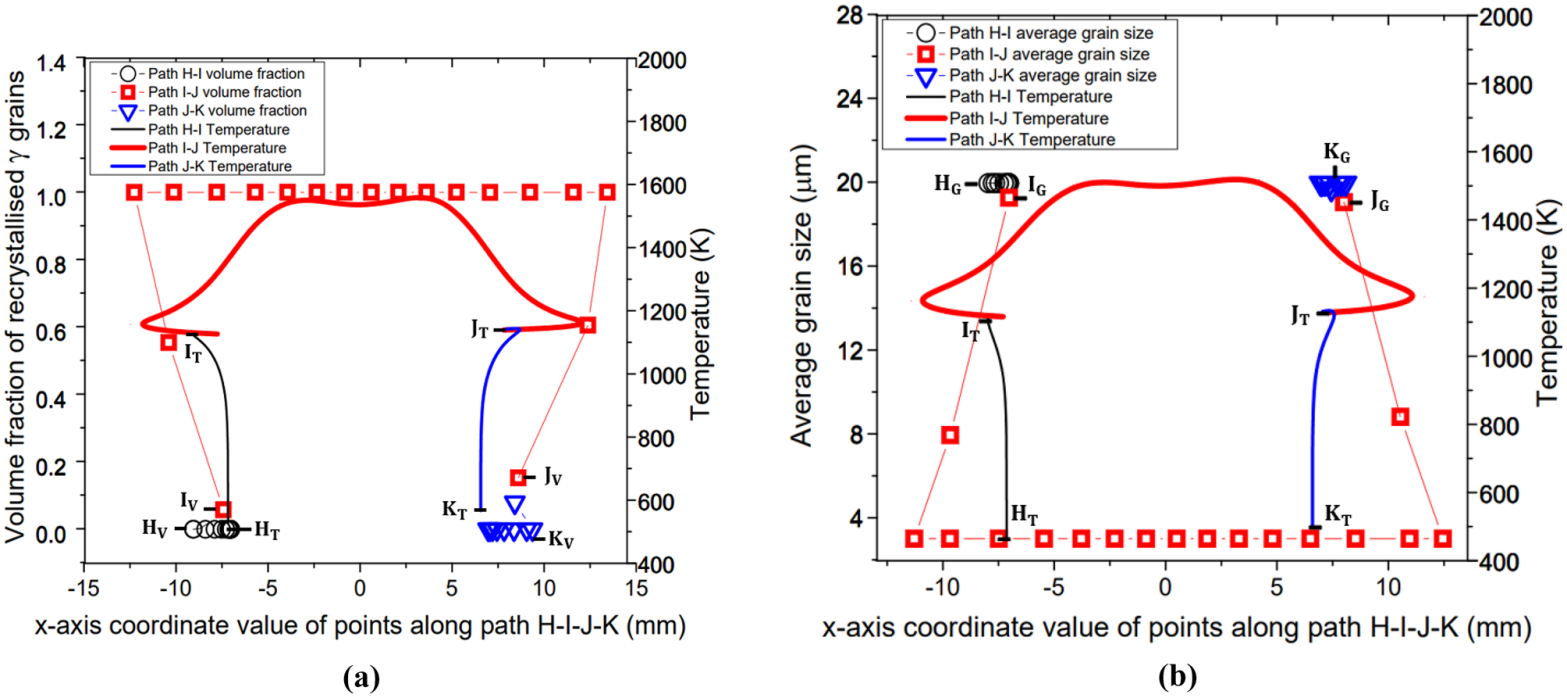
Computational modelling results of profile of (**a**) volume fraction of recrystallised *γ* grains and temperature (**b**) average size of *γ* grains and temperature along paths H─I, I─J, and J─K for LFW setup J13, with subscript ‘V’ for volume fraction, ‘T’ for temperature, and subscript ‘G’ for grain size

Figure 12a, b show the profiles of temperature (identified with subscript ‘T’), volume fraction of recrystallised *γ* grains (identified with subscript ‘V’), and average *γ* grain size (identified with subscript ‘G’) along each path H─I, I─J, and J─K for LFW setup J13 at 5.0 s of welding. The centre of friction interface of the bottom workpiece is at $x = 0$ mm according to the Cartesian coordinates (*X*–*Y* axes) of the 2D model.

In Fig. 12a, b, the profiles of volume fraction of recrystallised *γ* grains and average *γ* grain size, along paths H─I and J─K for setup J13 show that no DRX (and corresponding grain refinement) happened at the side surface of weld (along path H─I and path J─K) because the maximum temperature (1116 K) is considerably below the critical temperature for onset of DRX (1213 K). However, for the profile along path I─J, which includes the friction interface of the weld, *γ* grains are fully recrystallised resulting in significant grain refinement up to $x =\pm$ 5.4 mm relative to the centre of friction interface. This is due to the significantly elevated level of temperature along path I─J.

**Fig. 13:**
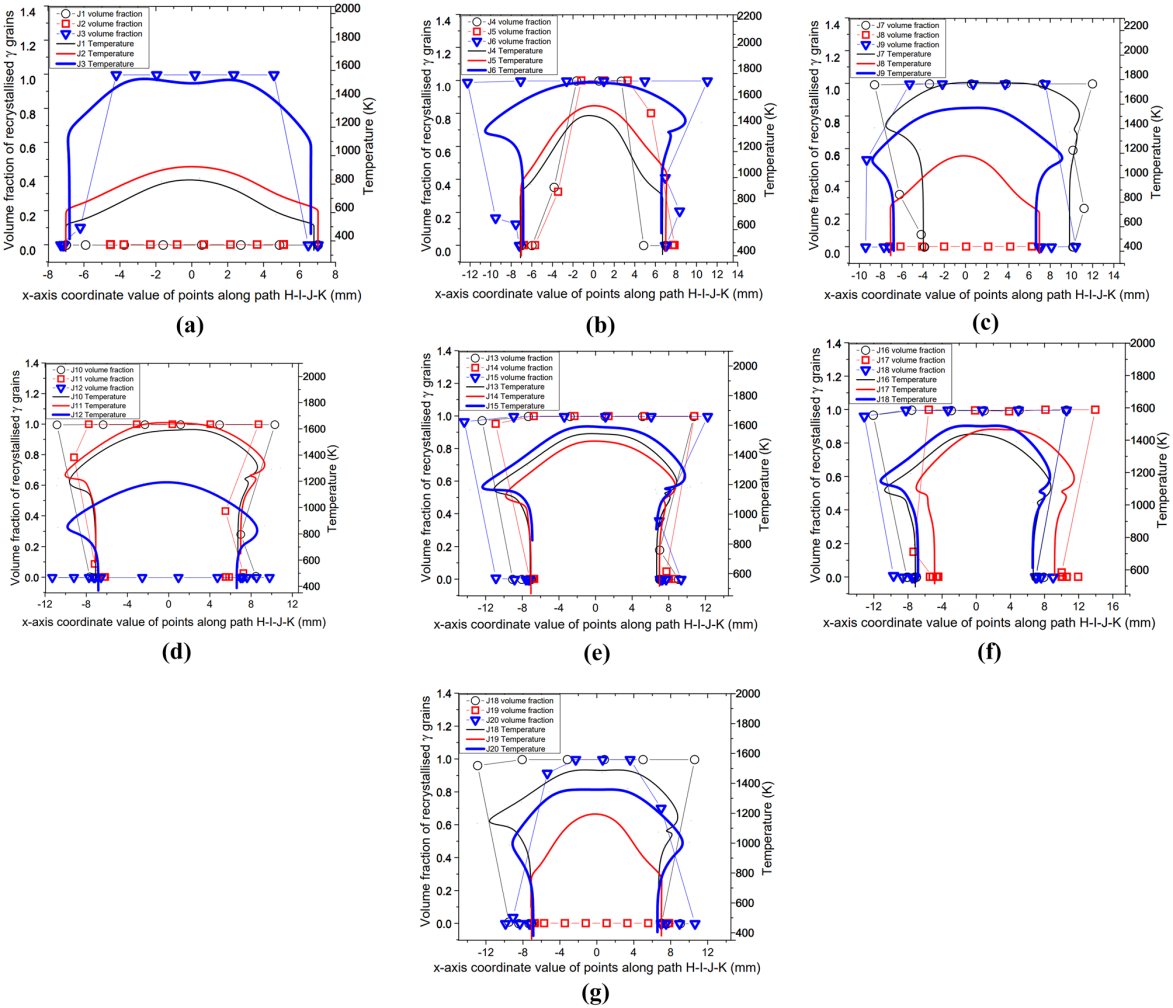
Computational modelling results of profile of volume fraction of recrystallised grains and temperature along the path H─I─J─K for LFW setups (**a**) J1, J2, and J3; (**b**) J4, J5, and J6; (**c**) J7, J8, and J9; (**d**) J10, J11, and J12; (**e**) J13, J14, and J15; (**f**) J16, J17, and J18; (**g**) J18, J19, and J20

**Fig. 14:**
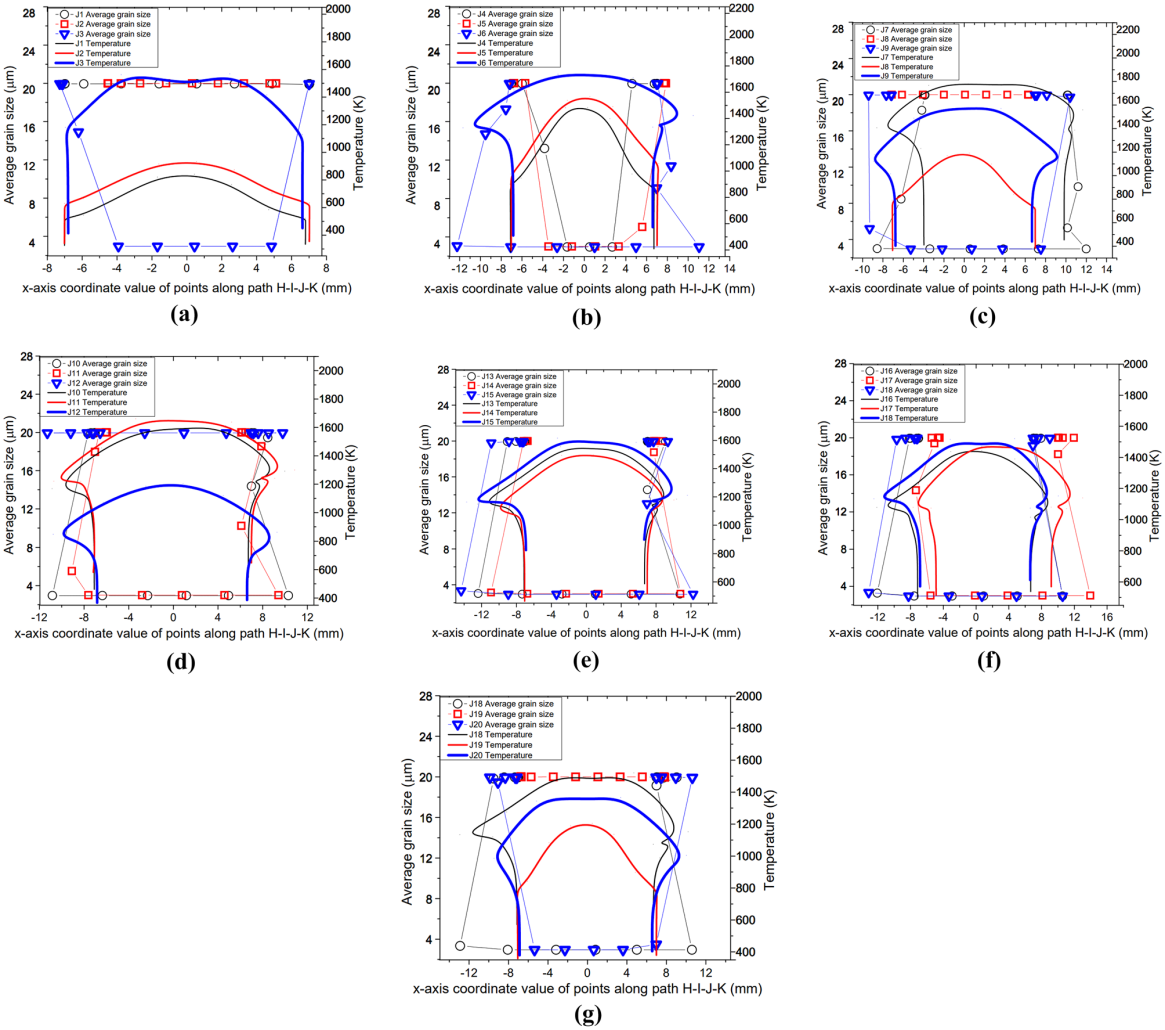
Computational modelling results of profile of average *γ* grain size and temperature along the path H─I─J─K for LFW setups (**a**) J1, J2, and J3; (**b**) J4, J5, and J6; (**c**) J7, J8, and J9; (**d**) J10, J11, and J12; (**e**) J13, J14, and J15; (**f**) J16, J17, and J18; (**g**) J18, J19, and J20

To achieve process parameter optimisation, the modelling results for setups J1 to J20 are herein presented following the same relationships as in setup J13 by relating the average *γ* grain size, volume fraction of recrystallised *γ* grains and temperature of weld. Figures 13a, g and 14a, g illustrate the profiles of volume fraction of recrystallised *γ* grains, average *γ* grain size, and temperature along the path H─I─J─K at 5.0 s of welding for all 20 different LFW setups. The 20 different LFW setups resulted in varying levels of weld temperature and microstructural evolution in terms of recrystallisation and grain refinement, as presented in this section and subsequent sections of this paper.

Figures 13a and 14a show that no DRX of *γ* grains occurred during LFW process of setup J1 and J2, because the maximum temperature is 850 K and 933 K, which is well below the critical temperature for onset of DRX (1213 K). For LFW setup J3, *γ* grains are fully recrystallised and refined within $x =\pm$ 5.8 mm relative to the centre of weld along the path H─I─J─K. The maximum temperature of setup J3 is 1585 K, which is below the IN718 liquidus temperature of 1633 K; hence, no remelting happened at the friction interface of weld.

For setups J1 and J2, there is low friction pressure ${p}_{f}=100\mathrm{ MPa}$, low oscillating frequency ${f}_{0}\le 20\mathrm{ Hz}$, small oscillating amplitude ${A}_{0}=2.5\mathrm{ mm}$, and correspondingly low average rubbing velocity of ${v}_{r}\le 200\mathrm{ mm}/\mathrm{s}$. Such setups could not result in significant plastic deformation, as discussed in the authors’ previous work [33, 34]. Although setup J3 is based on very low friction pressure ${p}_{f}=100\mathrm{ MPa}$, its frequency ${f}_{0}=40\mathrm{ Hz}$ and amplitude ${A}_{0}=3.3\mathrm{ mm}$ are at a very high level, resulting in significant weld deformation and DRX of *γ* grains. This is why significant grain refinement can be seen in Fig. 14a for setup J3.

Figures 13b–c and 14b–c show that setups J4, J5, J6, J7, and J9 resulted in fully recrystallised and significantly refined *γ* grains within $x =\pm$ 1.9 mm, $\pm$ 5.6 mm, $\pm$ 12.4 mm, $\pm$ 15.7 mm, and $\pm$ 8.7 mm, respectively, relative to the centre of friction interface. These setups have at least two LFW process parameters at a very high level, for instance, ${p}_{f}\ge 200\mathrm{ MPa}$, ${f}_{0}\ge 20\mathrm{ Hz}$, ${A}_{0}\ge 2.5\mathrm{ mm}$, and ${v}_{r}\ge 264\mathrm{ mm}/\mathrm{s}$. The maximum friction interface temperatures of these setups in Figs. 13b–c and 14b–c are below the liquidus temperature of IN718 (no remelting occurred) except for setup J7 (1791 K), which is based on two extremely high levels of process parameters ${f}_{0}=40\mathrm{ Hz}$ and ${A}_{0}=3.3\mathrm{ mm}$. Fully recrystallised and significantly refined grains can happen when running LFW using such extremely high levels of process parameters, which however can also cause extremely high weld temperature and excessive flash formation during LFW [20, 35, 40]. Although setup J3 is based on extremely high frequency and large amplitude like J7, it uses low friction pressure of 100 MPa, which does not cause a very high weld temperature that may exceed the liquidus temperature of IN718.

In Figs. 13c–g and 14c–g, the results of setups J8, J12, and J19 are similar to those of J1 and J2, where no DRX and no grain refinement happened, and weld deformation was very little. However, setups J10, J11, J13, J14, J15, J16, J17, J18, and J20 resulted in fully recrystallised and significantly refined grains up to $x =\pm$ 11.0 mm, $\pm$ 12.9 mm, $\pm$ 10.2 mm, $\pm$ 11.2 mm, $\pm$ 12.7 mm, $\pm$ 11.8 mm, $\pm$ 11.7 mm, $\pm$ 13.1 mm, and $\pm$ 5.3 mm, respectively, relative to the centre of friction interface. These setups have one process parameter at a low level, such as ${p}_{f}\le 300\mathrm{ MPa}$, ${f}_{0}\le 20\mathrm{ Hz}$, ${A}_{0}\le 2.5\mathrm{ mm}$, and ${v}_{r}\le 264\mathrm{ mm}/\mathrm{s}$ and the combination of at least two process parameters at very high levels. The maximum friction interface temperatures of these setups in Figs. 13c–g and 14c–g are below the liquidus temperature of IN718; thus, no remelting occurred during LFW. Path H─I─J─K was significantly elongated in the LFW setups that have ${f}_{0}\ge 30\mathrm{ Hz}$ and ${A}_{0}\ge 2.9\mathrm{ mm}$.

The overall results shown in Figs. 13a–g and 14a–g indicate that for different LFW setups, DRX and grain refinement did not happen at the sides of the bottom workpiece (such as approximately along paths H─I and J─K) due to relatively low temperature and plastic deformation. However, depending on the specified process parameters, temperature levels higher than the critical temperature for initiating DRX (~ 1213 K) were predicted near the centre of path I─J (on the friction interface). Irrespective of the friction pressure, no DRX, grain refinement, or significant material deformation of the weld was obtained when ${f}_{0}=15\mathrm{ Hz}$, ${A}_{0}=2.5\mathrm{ mm}$, and ${v}_{r}=150\mathrm{ mm}/\mathrm{s}$ (like setups J1, J8, J12, and J19). When friction pressure is low such as ${p}_{f}\le 200\mathrm{ MPa}$, either frequency ${f}_{0}\ge 30\mathrm{ Hz}$ or amplitude ${A}_{0}\ge 3.3\mathrm{ mm}$ needs to be at a very high level in order to cause significant DRX and grain refinement on the friction interface. Besides setups J1, J2, J8, J12, and J19, there is DRX happening either partially or completely in other LFW setups along the path H─I─J─K (mostly on path I─J of weld). It can be seen in the modelling results that partial or full DRX happens only when the temperature is higher than 1213 K.

When the LFW process parameters are all at high levels such that ${p}_{f}\ge 200\mathrm{ MPa}$, ${f}_{0}\ge 40\mathrm{ Hz}$, ${A}_{0}\ge 3.3\mathrm{ mm}$, and ${v}_{r}\ge 528\mathrm{ mm}/\mathrm{s}$, the maximum weld temperature can get higher than IN718 liquidus temperature, which indicates that remelting can occur at the friction interface. In terms of computational modelling, such extreme LFW process parameters can cause difficulties in dynamic remeshing of computational mesh during LFW process modelling [34, 41]. In terms of the practical LFW process, such extreme process parameters can result in excessive flash formation and axial shortening.

Figures 13a–g and 14a–g show that the weld material along path H─I─J─K was significantly elongated for setups using ${p}_{f}\ge 200\mathrm{ MPa}$, ${f}_{0}\ge 30\mathrm{ Hz}$, ${A}_{0}\ge 2.9\mathrm{ mm}$, and ${v}_{r}\ge 348\mathrm{ mm}/\mathrm{s}$ (like setups J7, J11, and J15). For the same level of frequency and amplitude, the region of material that gets fully recrystallised becomes larger when friction pressure increases from ${p}_{f}=100\mathrm{ MPa}$ to ${p}_{f}=600\mathrm{ MPa}$ (like the LFW setups J5, J10, J13, and J17). At the same level of friction pressure, for instance, 300 MPa or 400 MPa (like in LFW setups J8, J9, J10, J11, J12, J13, J14, and J15), the region of fully recrystallised material becomes larger when higher values of frequency (${f}_{0}\ge 25\mathrm{ Hz}$), amplitude (${A}_{0}\ge 2.9\mathrm{ mm}$), and average rubbing velocity ${(v}_{r}\ge 290\mathrm{ mm}/\mathrm{s})$ are employed. The level of pressure turns out to be a critical parameter because there is no DRX during LFW when ${p}_{f}\le 200\mathrm{ MPa}$ regardless of the different levels of frequency and amplitude that were tested in the computational modelling.

#### LFW process window

**Fig. 15:**
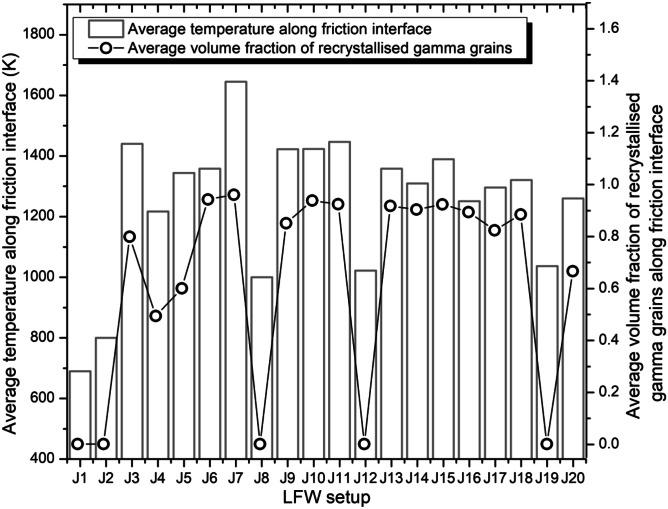
Computational modelling results of average temperature and average volume fraction of recrystallised *γ* grains along the friction interface at 5.0 s of welding for 20 different LFW setups

**Fig. 16:**
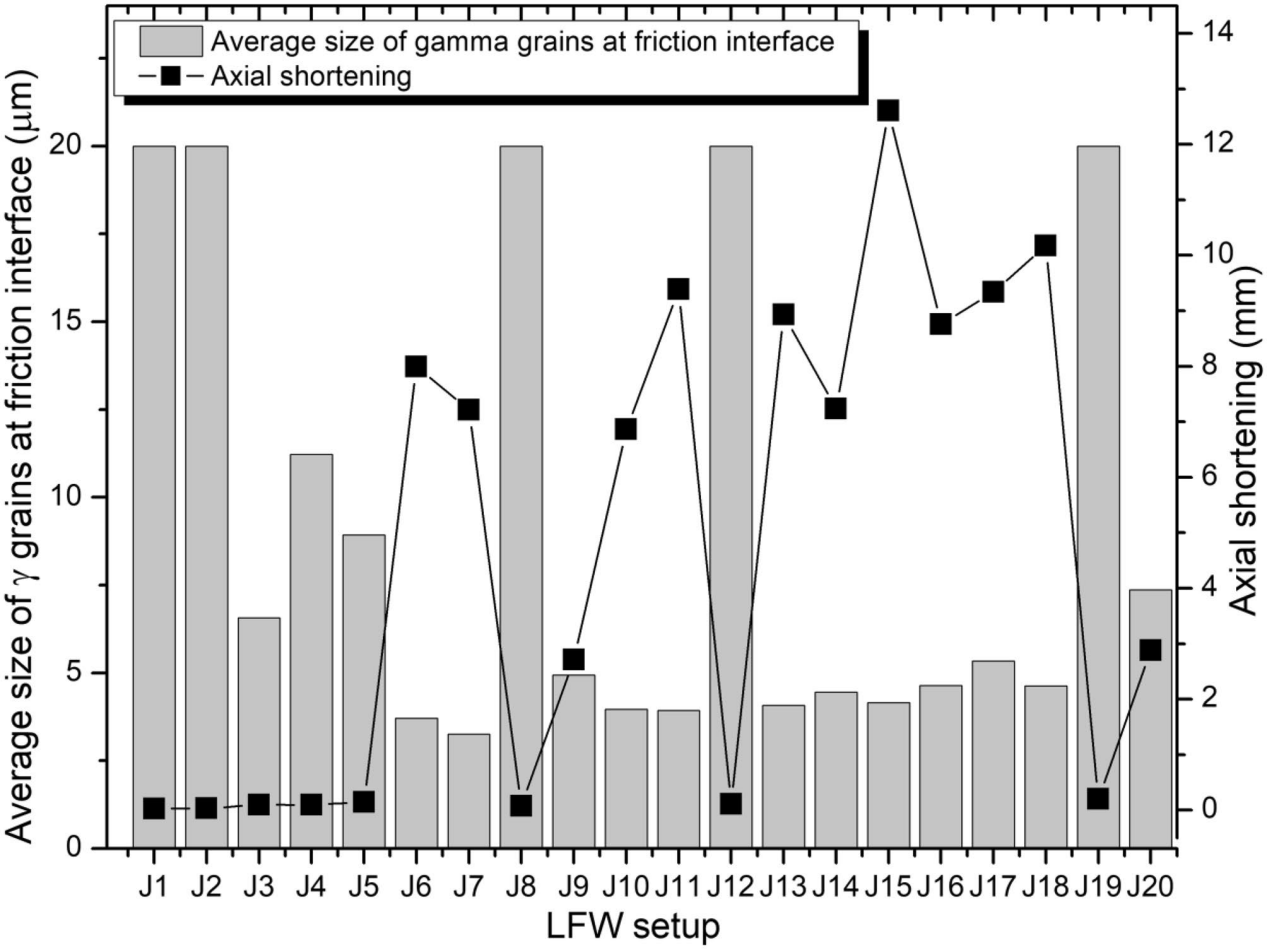
Computational modelling results of average *γ* grain size at friction interface and axial shortening of welds at 5.0 s of welding for 20 different LFW setups

To determine the LFW process window, the modelling results of weld temperature, average *γ* grain size, and axial shortening were considered for all 20 LFW setups. The modelling results of average temperature and average volume fraction of recrystallised *γ* grains at the friction interface along path I─J (bottom workpiece) at 5.0 s of welding for all 20 different LFW setups can be seen in Fig. 15. The modelling results of average *γ* grain size at the friction interface and axial shortening of top and bottom workpieces for all 20 different LFW setups at 5.0 s of welding can be seen in Fig. 16. Overall, for all 20 different LFW setups, the average volume fraction of recrystallised *γ* grains is high and the average *γ* grain size is small along path I─J when the average temperature of friction interface is high. For all 20 LFW, average temperature, average volume fraction of recrystallised *γ* grains, and average *γ* grain size at the friction interface are in the ranges of 691–1632 K, 0.01–95.0%, and 3.1–20.0 μm, respectively. No significant remelting occurred in any LFW simulation.

As shown in Fig. 16, axial shortening is low when ${p}_{f}=100\mathrm{ MPa}$ irrespective of frequency and amplitude. Additionally, axial shortening is low when using ${f}_{0}=15\mathrm{ Hz}$, ${A}_{0}=2.5\mathrm{ mm}$ (resulting in ${v}_{r}\ge 150\mathrm{ mm}/\mathrm{s}$), irrespective of pressure. Geng et al. recommended a critical (minimum) shortening length ${L}_{a}=4.8\mathrm{ mm}$, for achieving a reliable IN718 weld joint, that is satisfied by LFW setups J6, J7, J9 to J11, J13 to J18, and J20 [11]. All the setups that satisfied the critical shortening length also resulted in refined grains due to recrystallisation without material remelting at the friction interface.

**Fig. 17:**
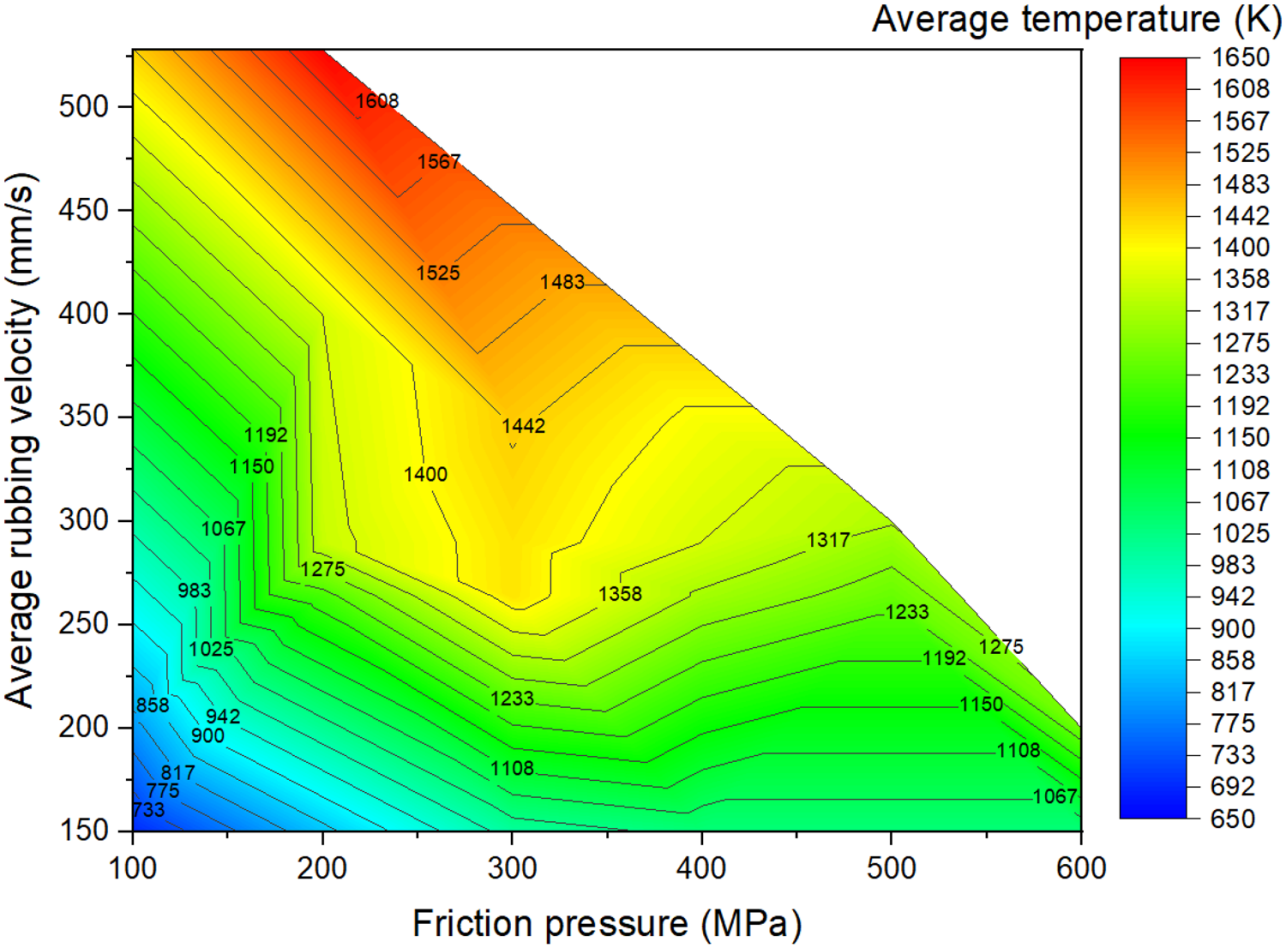
Process window in terms of average temperature at friction interface, average rubbing velocity, and friction pressure for 20 different LFW setups

**Fig. 18:**
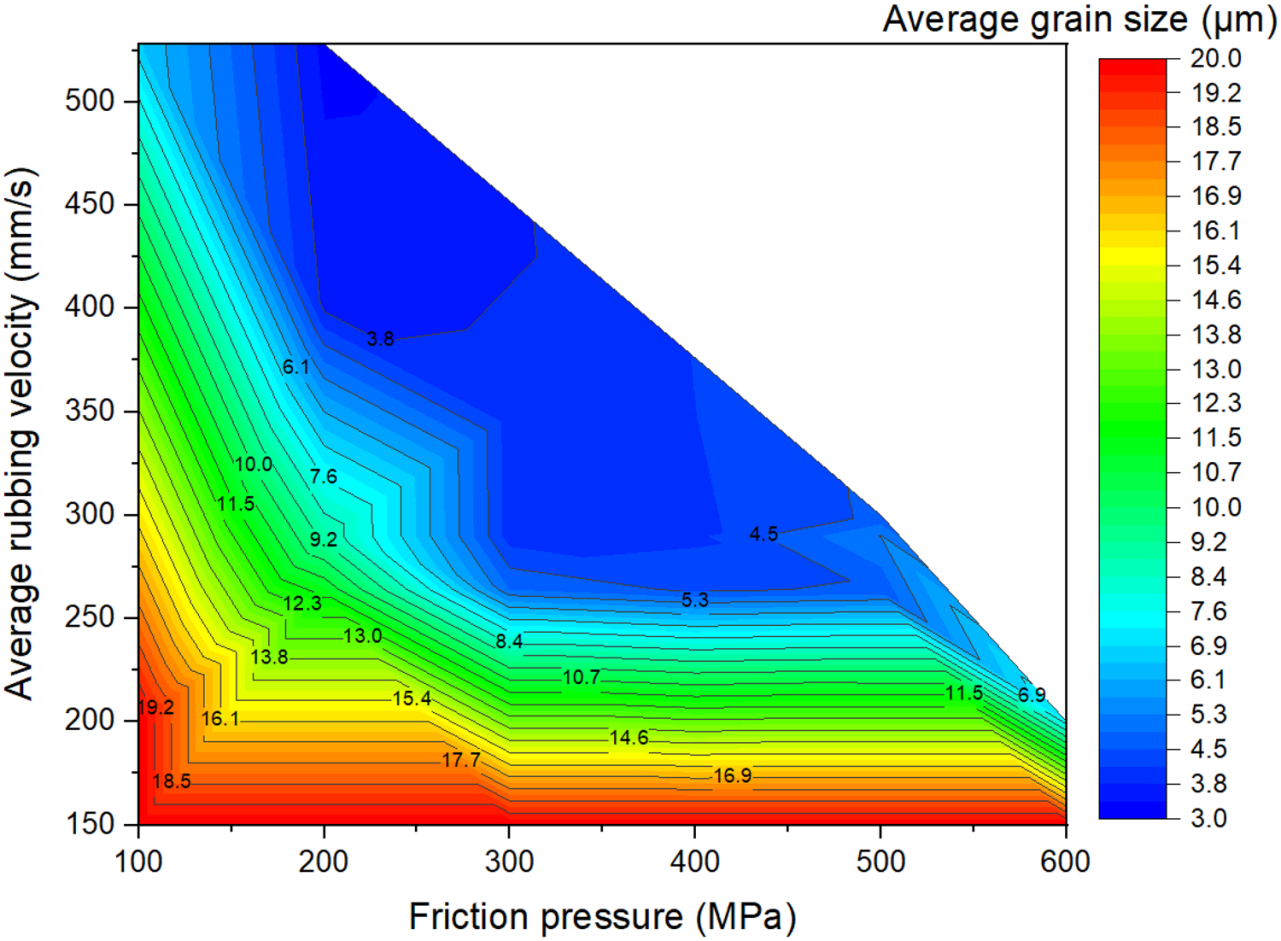
Process window in terms of average *γ* grain size at friction interface, average rubbing velocity, and friction pressure for 20 different LFW setups

**Fig. 19:**
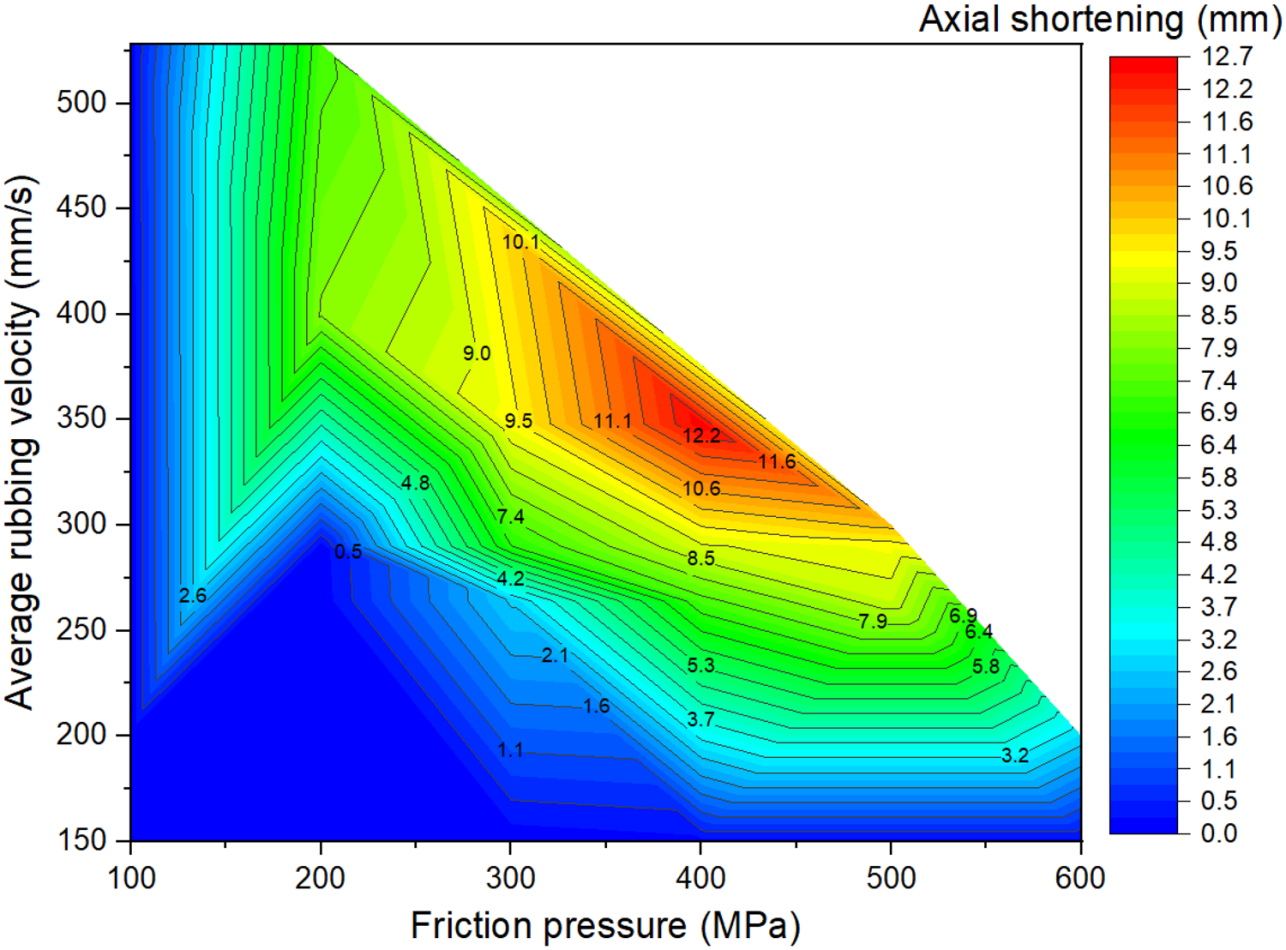
Process window in terms of axial shortening, average rubbing velocity, and friction pressure for 20 different LFW setups

Figures 17 to 19 show the LFW process windows that were created by using the computational modelling results (at 5.0 s of welding) of average rubbing velocity, friction pressure, average temperature along the friction interface, average *γ* grain size along the friction interface, and axial shortening for all 20 LFW setups. In the process windows, there are no computational modelling results for average rubbing velocity ${v}_{r}\ge 348\mathrm{ mm}/\mathrm{s}$ and friction pressure ${p}_{f}\ge 400\mathrm{ MPa}$, because such extreme process parameters caused excessive distortion of computational mesh and therefore corresponding computational simulation of related LFW process (at such extreme level of process parameters) could not be successfully completed.

In Fig. 17, it can be seen that high rubbing velocity results in high weld temperature, regardless of the different levels of friction pressure that were tested in the 20 different setups. This indicates that average rubbing velocity has a much more dominant influence on energy input for LFW than friction pressure. In Figs. 18 and 19, it can be seen that a high level of axial shortening and very significant grain refinement (due to DRX) can happen when there is a combination of high rubbing velocity and high friction pressure, for instance, when the friction pressure is approximately at the level of 400 MPa, and rubbing velocity approximately at the level of 350 mm/s. Geng et al. attributed sufficient axial shortening and fine *γ* grains to good weld bonding quality as well as good strength and hardness of the weld [11].

It can be concluded from the computational modelling results shown in Figs. 13 to 19 that high levels of friction pressure (≥ 200 MPa), oscillating frequency (≥ 25 Hz), oscillating amplitude (≥ 2.9 mm), and average rubbing velocity (≥ 290 mm/s) result in high average temperature at the friction interface (≥ 1453 K), very small *γ* grain size (≤ 5.6 μm) due to DRX, and high axial shortening of the overall weld (≥ 5.8 mm). Friction pressure was found to be the most influential process parameter and the least influential process parameter is oscillating amplitude, for determining average temperature, average volume fraction of recrystallised *γ* grains, and average *γ* grain size at the friction interface as well as axial shortening of welds.

## Conclusions

In this study, integrated multiphysics computational modelling for LFW process was developed by sequentially coupling a thermomechanical model with a microstructural model. Heat transfer, elastic and plastic deformation of weld, and dynamic recrystallisation are in the modelling, which predicts the results of LFW of IN718 in terms of such as weld temperature, plastic strain, volume fraction of recrystallised *γ* grains, and resulting *γ* grain size as well as axial shortening of the overall weld. For the first time, an integrated multiphysics computational modelling has been developed for predicting LFW of IN718 at the scale of the overall weld. The integrated computational modelling was verified by comparing its modelling results of *γ* grain size profile of weld with related experimental results of other researchers [11].

The influence of process parameters (pressure, frequency, amplitude, and average rubbing velocity) on predicting weld temperature, axial shortening, volume fraction of recrystallised *γ* grains, and average *γ* grain size of IN718 LFW welds was systematically analyzed by using 20 different LFW setups in the computational modelling. Friction pressure (≥ 200 MPa) was found to be the most significant process parameter influencing the recrystallisation of *γ* grains, as well as weld temperature and axial shortening. High friction pressure results in high temperature and high strain rate, which significantly drive dynamic recrystallisation of *γ* grains around the friction interface of weld during LFW. Frequency and amplitude showed less significant influence compared to friction pressure, and amplitude turns out to be the least influential LFW process parameter. The related LFW process windows (Figs. 17 to 19) consistently show that at least two LFW process parameters must be simultaneously at a very high level in order to achieve sufficient axial shortening of overall weld (≥ 5.8 mm) and significantly refined *γ* grains (≤ 5.6 μm) around the friction interface. The integrated computational modelling can effectively and efficiently help the manufacturing industry to optimise the design of LFW process parameters.

## Data Availability

The data used to support the findings of this study are available from the funding source on demand.

## Acronyms and Greek

DRX: Dynamic Recrystallisation
IN718: Inconel 718
JMAK: Johnson-Mehl-Avrami-Kolmogorov
LFW: Linear friction welding
Ni: Nickel
$${\alpha }_{w}$$: Thermal expansion [K^−1^]
$$\delta$$: Delta phase
$$\varepsilon$$: Plastic strain
$$\dot{\varepsilon }$$: Strain rate [s^−1^]
$${\varepsilon }_{c}$$: Critical strain
$${\varepsilon }_{p}$$: Peak strain
${\varepsilon }_{0.5}$: Plastic strain for 50% recrystallised volume fraction
${\varepsilon }_{c1}$, ${\varepsilon }_{b1}$: IN718 DRX constants
$\eta$: Efficiency of converting mechanical energy to heat energy
$$\gamma$$: Gamma grain
${\gamma ^\prime}$: Gamma prime phase
${\gamma ^{\prime\prime}}$: Gamma double prime phase
$$\lambda$$: Thermal conductivity [W·m^−1^ K^−1^]
$\mu$: Coulomb’s friction coefficient
$$\nu$$: Poisson’s ratio
$$\phi$$: Emissivity
$$\rho$$: Density [kg·m^−3^]
${\sigma }_{e}$: Effective stress [MPa]
${\sigma }_{s}$: Shear stress [MPa]
${\sigma }_{y}$: Yield stress [MPa]
${\tau }_{fr}$: Frictional shear stress [MPa]
$\theta$: Stefan–Boltzmann constant [W·m^−^^2^ K^−^^4^]
VUHARD: Abaqus/Explicit user-hardening subroutine

## Abbreviations

${A}_{0}$: Oscillating amplitude [mm]
$A(\varepsilon )$, $n(\varepsilon )$, $\alpha (\varepsilon )$: IN718 material constants
${c}_{p}$: Specific heat capacity [J·kg^−1^ K^−1^]
$d_0$: Initial γ grain size [μm]
$d_{ave}$: Average γ grain size [μm]
$d_{DRX}$: Recrystallised γ grain size [μm]
$F$: Body force vector per unit mass [N·kg^−1^]
${f}_{0}$: Oscillating frequency [Hz]
${g}_{c1}$, ${g}_{c2}$, ${g}_{c3}$, ${g}_{l1}$, ${g}_{l2}$, ${g}_{l3}$, ${n}_{b1}$, ${n}_{c1}$: IN718 DRX constants
${h}_{k}$: Convective heat transfer coefficient [W·m^−2^ K^−1^]
${L}_{a}$: Axial shortening [mm]
$n$: Surface area of workpiece [m^2]^
$${n}_{d}$$: Avrami constant
${p}_{f}$: Friction pressure [MPa]
$Q(\varepsilon )$: Deformation activation energy [J·mol^−1^]
$R$: Gas constant [J·mol^−1^ K^−1^]
$$t$$: Time [s]
${t}_{k}$: Current step time [s]
${t}_{k-1}$: Previous step time [s]
$$T$$: Weld temperature [K]
$${T}_{c}$$: Ambient temperature [K]
${T}_{w}$: Wall (boundary) temperature [K]
${v}_{s}$: Slip velocity [mm·s^−1^]
${v}_{r}$: Average rubbing velocity [mm·s^−1^]
${X}_{DRX}$: Recrystallised volume fraction
$Z$: Zener-Hollomon parameter [s^−^^1^]
